# Heterologous expression of Arabidopsis laccase2, laccase4 and peroxidase52 driven under developing xylem specific promoter DX15 improves saccharification in populus

**DOI:** 10.1186/s13068-023-02452-7

**Published:** 2024-01-13

**Authors:** Yogesh K. Ahlawat, Ajaya K. Biswal, Sarahani Harun, Anne E. Harman-Ware, Crissa Doeppke, Nisha Sharma, Chandrashekhar P. Joshi, Bertrand B. Hankoua

**Affiliations:** 1 Department of Biological Sciences, Michigan Technological University, Houghton, MI 49931 USA; 2 Department of Biochemistry and Molecular Biology, University of Georgia, Athens, GA30602 USA; 3 Complex Carbohydrate Research Center, University of Georgia, Athens, GA30602 USA; 4 Centre for Bioinformatics Research, Institute of Systems Biology (INBIOSIS), Universiti Kebangsaan Malaysia, UKM Bangi, Selangor Malaysia; 5 Renewable Resources and Enabling Sciences Center, National Renewable Energy Laboratory, Golden, CO 80401 USA; 6 Microbiology Section, Department of Basic Science, Dr. Y.S Parmar University of Horticulture and Forestry, Nauni, Solan, Himachal Pradesh India; 7 Human Ecology Department, College of Agriculture, Science, and Technology (CAST), Food Science and Biotechnology Program, 1200 N. DuPont Highway, Dover, DE 19901 USA

**Keywords:** Heterologous expression, Laccases, Peroxidases, Developing xylem, Saccharification, Secondary cell walls, Arabidopsis, Poplars, Bioethanol

## Abstract

**Background:**

Secondary cell wall holds considerable potential as it has gained immense momentum to replace the lignocellulosic feedstock into fuels. Lignin one of the components of secondary cell wall tightly holds the polysaccharides thereby enhancing the recalcitrance and complexity in the biomass. Laccases (LAC) and peroxidases (PRX) are the major phenyl-oxidases playing key functions during the polymerization of monolignols into lignin. Yet, the functions of laccase and peroxidases gene families remained largely unknown. Hence, the objective of this conducted study is to understand the role of specific LAC and PRX in Populus wood formation and to further investigate how the altered Lac and Prx expression affects biomass recalcitrance and plant growth. This study of heterologous expression of Arabidopsis Lac and Prx genes was conducted in poplar to avoid any otherwise occurring co-suppression mechanism during the homologous overexpression of highly expressed native genes. In the pursuit of optimizing lignocellulosic biomass for biofuel production, the present study focuses on harnessing the enzymatic potential of *Arabidopsis thaliana* Laccase2, Laccase4, and Peroxidase52 through heterologous expression.

**Results:**

We overexpressed selected Arabidopsis laccase2 (AtLac2), laccase4 (AtLac4), and peroxidase52 (AtPrx52) genes, based on their high transcript expression respective to the differentiating xylem tissues in the stem, in hybrid poplar (cv. 717) expressed under the developing xylem tissue-specific promoter, DX15 characterized the transgenic populus for the investigation of growth phenotypes and recalcitrance efficiency. Bioinformatics analyses conducted on AtLac2 and AtLac4 and AtPrx52, revealed the evolutionary relationship between the laccase gene and peroxidase gene homologs, respectively. Transgenic poplar plant lines overexpressing the AtLac2 gene (AtLac2-OE) showed an increase in plant height without a change in biomass yield as compared to the controls; whereas, AtLac4-OE and AtPrx52-OE transgenic lines did not show any such observable growth phenotypes compared to their respective controls. The changes in the levels of lignin content and S/G ratios in the transgenic poplar resulted in a significant increase in the saccharification efficiency as compared to the control plants.

**Conclusions:**

Overall, saccharification efficiency was increased by 35–50%, 21–42%, and 8–39% in AtLac2-OE, AtLac4-OE, and AtPrx52-OE transgenic poplar lines, respectively, as compared to their controls. Moreover, the bioengineered plants maintained normal growth and development, underscoring the feasibility of this approach for biomass improvement without compromising overall plant fitness. This study also sheds light on the potential of exploiting regulatory elements of DX15 to drive targeted expression of lignin-modifying enzymes, thereby providing a promising avenue for tailoring biomass for improved biofuel production. These findings contribute to the growing body of knowledge in synthetic biology and plant biotechnology, offering a sustainable solution to address the challenges associated with lignocellulosic biomass recalcitrance.

**Supplementary Information:**

The online version contains supplementary material available at 10.1186/s13068-023-02452-7.

## Introduction

Second generation biofuels exploits lignocellulosic biomass produced from the secondary cell wall biosynthetic components such as cellulose, hemicellulose, and lignin [1]. However, complex and recalcitrant cell wall led to the use of pretreatment technologies, enzymatic saccharification, and fermentation. Plant cell walls are composed of various polymers, forming the structural framework that gives a plant cell a specific shape and reinforcement. The polysaccharides have a strong potential for providing raw materials for bioethanol, however lignin, a major polyphenolic polymer, impedes the release of free sugars from the lignocellulosic biomass. Therefore, it has been suggested that lignin plays an important role in saccharification by facilitating microbial fermentation. Three types of hydroxycinnamyl units (i.e., monomeric units of lignin, also known as monolignols, namely, sinapyl, coniferyl, and p-coumaryl alcohols, which are synthesized via phenylpropanoid pathway from phenylalanine determine the metabolic influx of polyphenols to plant cell walls. Lignification occurs in fibers and tracheary elements of vascular plants thereby strengthening their vascular system which protects the plants from any biotic or abiotic stress under adverse conditions [2]. Lignification is largely controlled and regulated by multigene families, similar expression patterns and functional redundancy possessed by laccases and peroxidases [3]. It is believed that PRX oxidizes the substrates using hydrogen peroxide as the electron acceptor and are encoded by 73, 93, and 138 genes in Arabidopsis [4], Populus [5], and rice [6], respectively. Mostly all PRXs oxidize coniferyl units but sinapyl units are only oxidized by a selective PRX and other phenyloxidases [7]. Class I and II PRXs are involved in lignin degradation and secretory function whereas class III PRXs are mainly involved in lignin biosynthesis. Class III PRX enzymes have been well studied in tobacco [8], Arabidopsis [9] Zinnia elegans [10], and many angiosperms and gymnosperms species [11]. Microarray studies in Zinnia elegans at different stages of TE development showed that Lac and Prx genes were specifically expressed in lignifying TE cells whereas monolignol biosynthetic genes were found to be expressed in both lignifying TE and non-lignifying TE elements suggesting that hydrogen peroxide and monolignols were supplied by the parenchyma cells in the culture [12]. Several PRXs have been shown to have a role in the lignification process in wood [13]. Since the poplar genome sequence is available now, it could provide important information regarding lignin-specific genes in trees to better understand the saccharification process in the woody biomass [14]. Peroxidase, PRX specific database, showed information of 105 Class III PRXs from plants [11] but analyzing functions of all the genes by reverse genetic studies would be a challenging task. Despite a lot of ongoing research in recent years, we still have limited knowledge about the role of PRXs in xylem lignification and saccharification process. [15] confirmed the role of PRX4 in S-lignin formation. Downregulation of AtPrx4 transcripts resulted in 37% decrease in lignin content but S/G ratio was decreased from 0.65 to 0.48. Similarly, AtPrx72 is involved in lignification and its transgenic suppression resulted in the thinning of interfascicular fibers. Downregulation of PRX TP60 in tobacco showed 50% reduction in lignin [8]. Lignin content was decreased by about 20% in transgenic aspen lines by down-regulation of PrxA3a [16]. All these results indicated that class III PRXs are involved in lignin biosynthesis. Most PRX genes have shown maximum expression at specific locations, i.e., xylem tissue where maximal lignification occurs. However, identification of exact functions of all class III PRXs in lignification is still unclear. Therefore, functional studies are required to provide answers to elucidate functions of various Prx genes.

The Laccase (LAC) is a family of multicopper oxidases which catalyze one electron oxidation of wide variety of substrates. LACs mainly oxidize ortho- and para-phenols and even monophenols into free radicals reducing the molecular oxygen to water during the process [17]. The possible functions of LACs in lignification in tree species has been reported [18]. There are 17 Lac genes in Arabidopsis but only 8 showed maximum expression along the inflorescence axis in the xylem-specific regions [19]. Laccases and peroxidases have been reported to play an important role in lignification process during the development of secondary xylem [20]. Double mutant of lac5lac8 in brachypodium showed 20–30% decrease in lignin content with a glucose increase by 140% compared to wild type whereas single mutant in brachypodium exhibited 10% decrease in lignin content [21]. This suggested that laccases act on stem lignification and therefore becomes a potential target to reduce the cell wall recalcitrance for enhancing saccharification [22]. Recently, [23] reported overexpression and knockdown of KNAT7 transcription factor in poplars where 22–53% saccharification yield and 5–12% S/G ratios were improved in the transgenic lines as compared to controls describing as one of the ways for increasing sugar yield for described as a tool to be potentially used in the application of bioethanol production. The precise role of LAC and PRX in improving saccharification efficiency is still unclear and therefore transgenic approaches may provide insights into exploring better ways to improve saccharification of woody biomass [24, 25].

For this conducted study, we chose LAC2, LAC4 and PRX52 genes as they exhibited highest transcript expression in the stem differentiating xylem (SDX) region where the maximum biomass production is expected. All three candidate genes have never been previously used for any transgenic studies targeting hybrid poplars species. Therefore, we attempted to investigate the specific functions of Arabidopsis LAC2, LAC4 and PRX52 in hybrid poplars using transgenic approaches. We also conducted a comprehensive bioinformatics study to elucidate the functions and evolutionary relationships of AtLAC2, AtLAC4 and AtPRX52 in silico. We generated transgenic poplar lines by overexpressing LAC2, LAC4 and PRX52 in poplar tissues using specific promoter DX15 to observe the effect of such transgenic manipulations on SCW. We performed heterologous expression of AtLac’s and AtPrx in poplar to avoid any otherwise naturally occurring co-suppression mechanism during the overexpression of native genes, routinely occurring due to high sequence homology or high copy number. Through this study, we understood how alteration in the SCW formation affects the cell wall recalcitrance, biochemical properties and saccharification efficiency of lignocellulosic biomass in hybrid poplar species. The objective of the conducted research is to engineer plants for better biofuel production or other applications related to biomass and feedstock utilization. The overexpression of lignin-modifying enzymes like laccases and peroxidases driven in specific tissues can lead to changes in the plant’s cell wall composition, potentially making it easier to extract sugars for biofuel production. It's also a strategy to reduce the recalcitrance of plant biomass, and therefore making it more amenable to industrial processes.

## Results

### Relative transcript expression of AtLac2, AtLac4 and AtPrx52 genes in transgenic poplars through RT-PCR

**Fig. 1:**
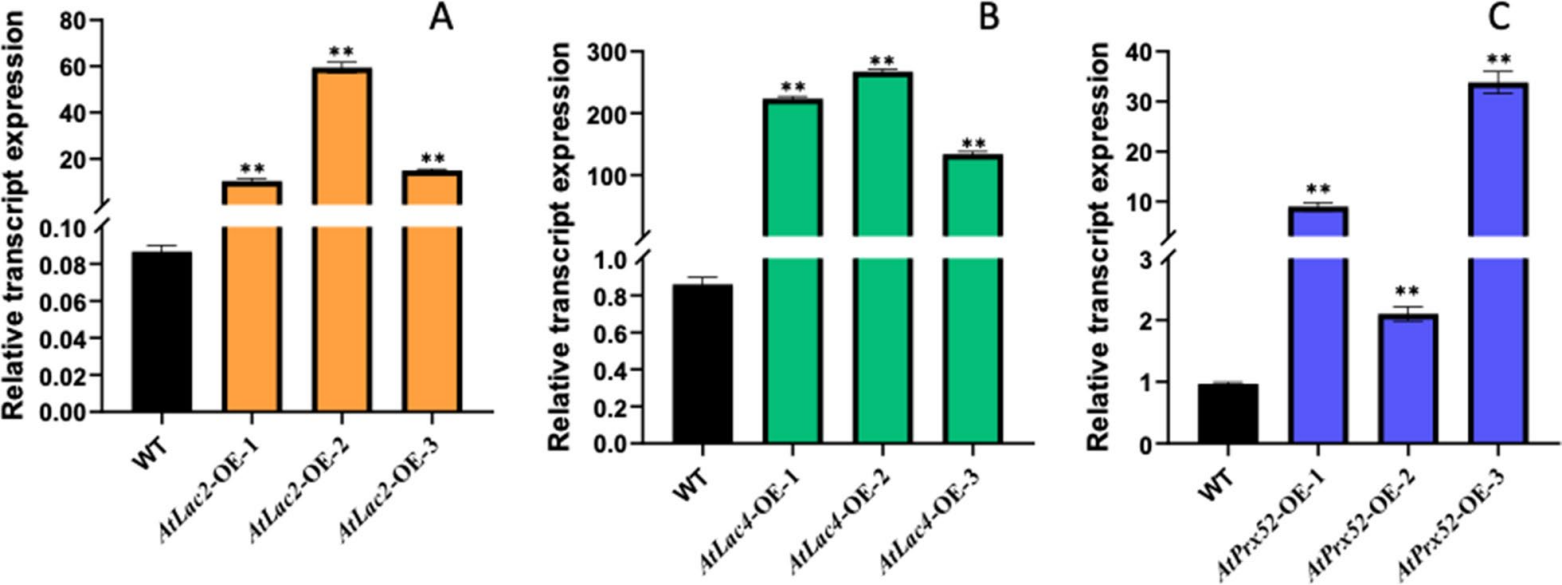
Transcript expression of *AtLac2-OE, AtLac4-OE* and *AtPrx52-OE* genes in poplar using real-time PCR. **a** Transcript expression in three independent lines expressing *AtLac2-OE*; **b** transcript expression in three independent lines expressing *AtLac4-OE*, **c** transcript expression in three independent lines expressing *AtPrx52-OE*. Wild type plants (control) did not show any expression for their respective transcripts in transgenic lines as expected. Transgene expression in all the transgenic lines is highly significant as compared to control (*P < 0.05, **P < 0.01, ***P < 0.001)

We tested three independent transgenic lines AtLac2-OE AtLac4-OE, and AtPrx52-OE for their transcript relative expression of and based on the highest transcript expressing lines were selected for phenotypic studies, enzyme assays and wood biochemical properties like lignin content and saccharification efficiency. From Fig. 1A–C, it is evident that the transcript expression of AtLac2-OE (Fig. 1A), AtLac4-OE (Fig. 1b), and AtPrx52-OE (Fig. 1C) is highly significant than control whereas no expression in control was observed as expected.

### Total enzyme activity for laccases and peroxidases in transgenic poplars

**Fig. 2:**
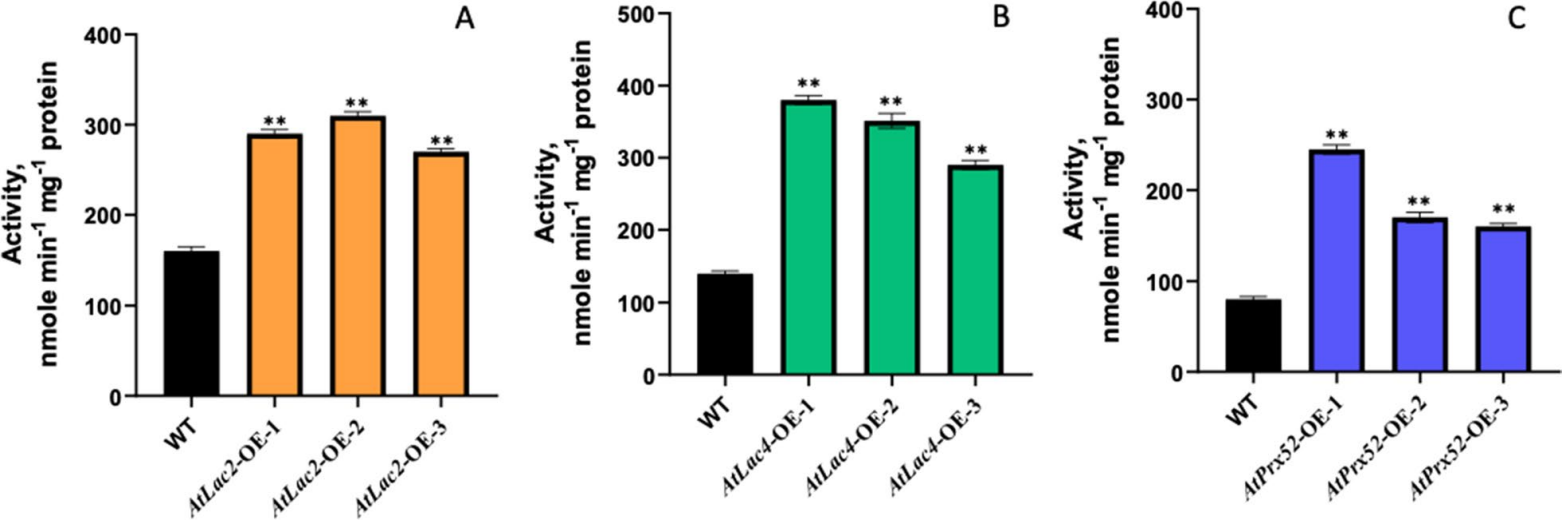
Estimation of LAC and PRX activity in transgenic poplar lines using stem differentiating xylem (SDX) extracts. **a** Total LAC activity in AtLac2-OE transgenic lines, **b** total LAC activity in AtLac4-OE transgenic lines, **c** total PRX activity in AtPrx52-OE transgenic lines. Significant differences were observed as compared to control (*P* < 0.0002)

Following the quantitative expression studies, total LAC and PRX activity determination was performed using ABTS (in case of laccase activity) and guaiacol (in case of peroxidase assay) as a substrate from the stem differentiating xylem extracts. Enzyme activity was recorded for a period of five minutes using extract from differentiating xylem extracts of *AtLac2-OE, AtLac4-OE* and *AtPrx52-OE* transgenic lines with each activity measured in triplicates. Data showed significant increase in total enzyme activity observed from all extracts derived from all the transgenic lines compared to extract obtained from non-transgenic control (Fig. 2A–C). Furthermore, observed increase in enzyme activity correlated with increase in respective transcript expression according to the gene expression studies (Fig. 1A–C).

### Effect of OE- AtLac2, AtLac4 and AtPrx52 on the biomass

**Fig. 3:**
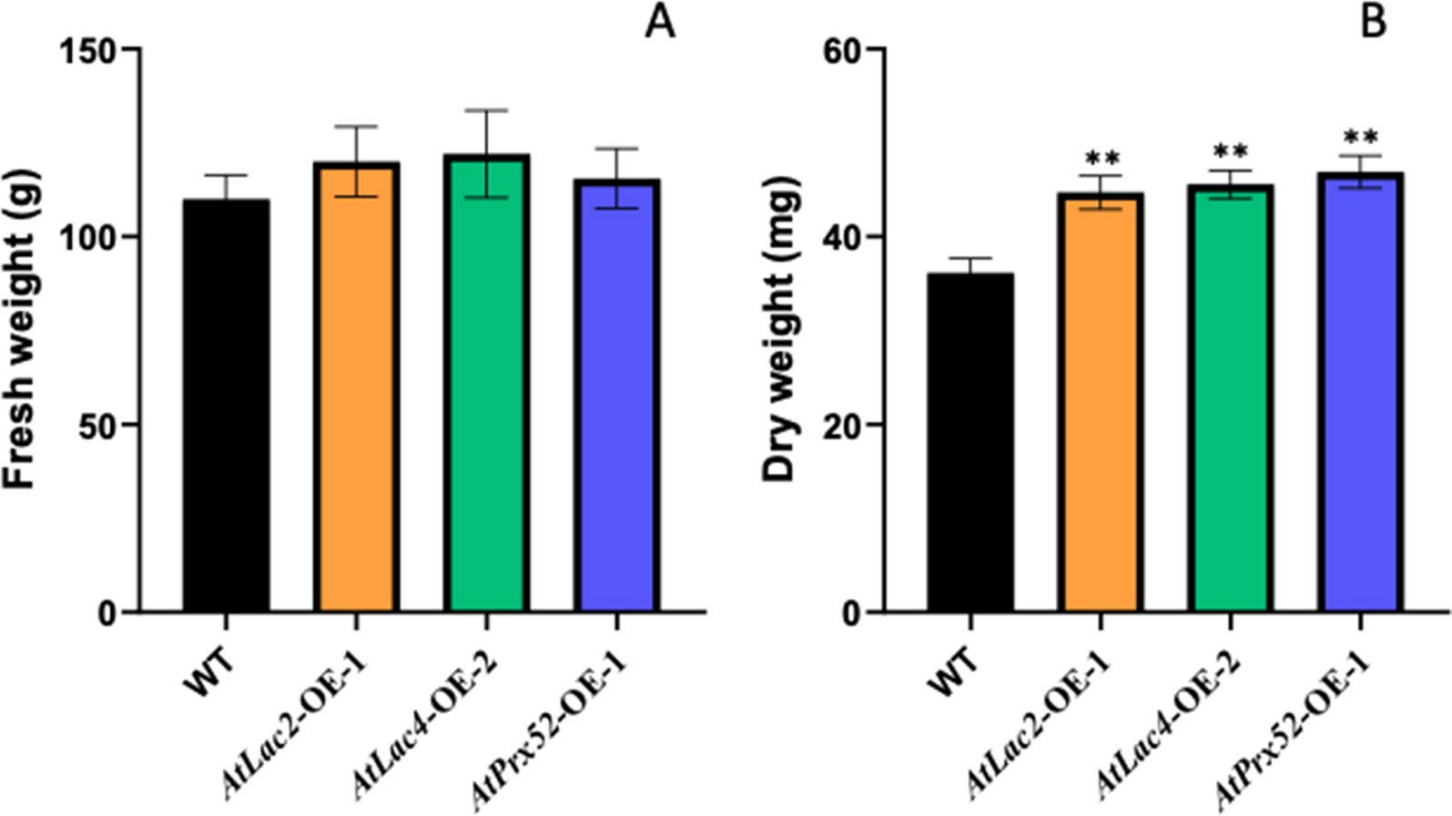
Fresh and dry biomass weight measurements of Control (wild type), transgenic lines poplar line (AtLac2-OE, AtLac4-OE, AtPrx52-OE). Fresh and dry biomass was measured by averaging the biological replicates from three transgenic lines of each gene construct and control. Significant (in case of dry weight) as compared to wild type (*P* < 0.01)

All fresh leaves from each of the non-transgenic control and transgenic plants expressing *AtLac2-OE, AtLac4-OE* and *AtPrx52-OE* genes were dried prior to measuring biomass characteristics. Total biomass data were scored as average of three independent transgenic lines for each gene. As shown in Fig. 3, no significant differences were observed in terms of fresh weight (Fig. 3A) across all expressers and however, dry biomass weight for each of the expressers was significantly higher than the respective control plants (WT) (Fig. 3B).

### Lignin content and S/G ratio changes in AtLac2-OE, AtLac4-OE and AtPrx52-OE lines

**Fig. 4:**
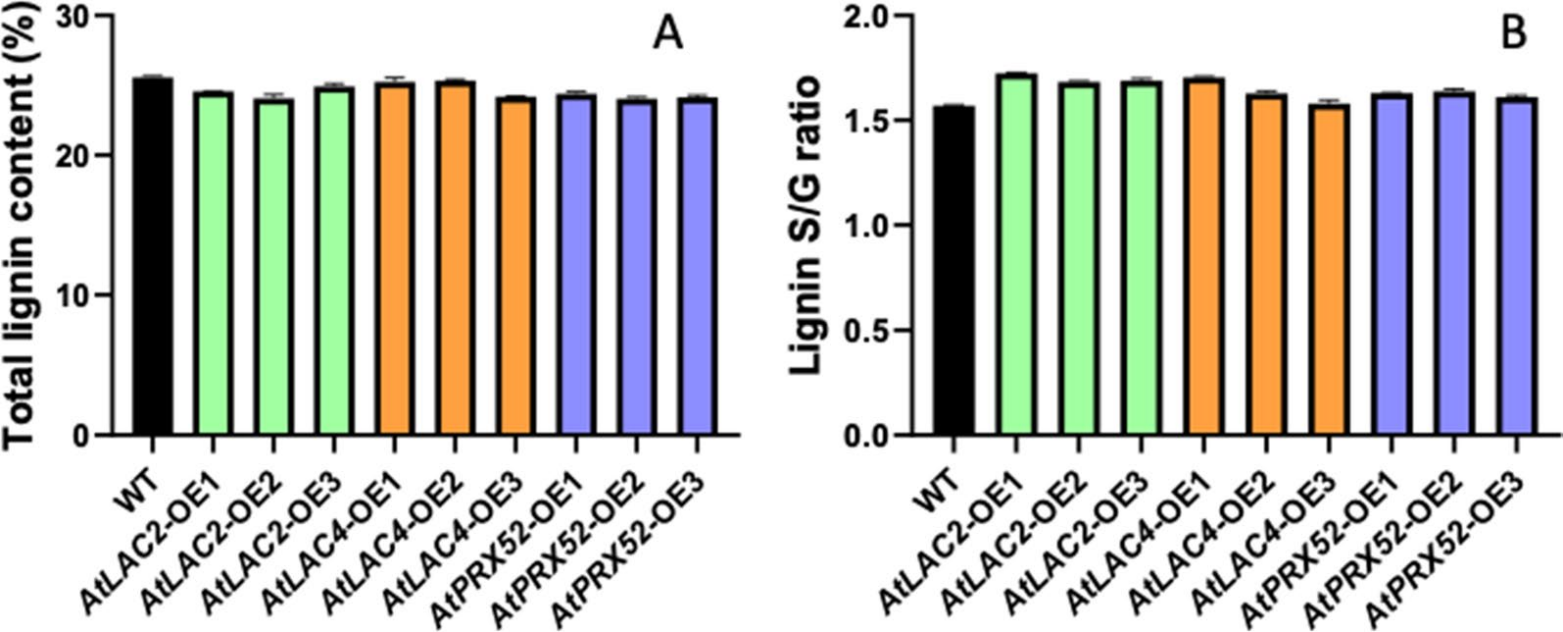
Lignin analysis for transgenic plants expressing *AtLac2-OE, AtLac4-OE* and *AtPrx52-OE* transgenic lines: **a** lignin content for *AtLac2-OE, AtLac4-OE* and *AtPrx52-OE* transgenic lines in poplar. **b** S/G ratio measurements for *AtLac2-OE, AtLac4-OE* and *AtPrx52-OE* transgenic lines in poplar. Significant as compared to wild type (*P* < 0.01)

The lignin content was measured using PyMBMS method at NREL and analysis was carried out in duplicate for each of the three samples of AtLac2-OE, AtLac4-OE and AtPrx52-OE lines including non-transformed poplar control. Transgenic poplar lines with increased transgene expression were further analyzed for lignin content. In Fig. 4A, lignin content decreased by 2–5% in AtLac2-OE and AtLac4-OE whereas 4–6% decrease was observed in line AtPrx52-OE. In Fig. 4B, the S/G ratio showed overall increase by 7–10% in AtLac2-OE, 4–9% in AtLac4-OE, and 3–4% increase AtPrx52-OE. Typically, S/G ratio provides the proportion of Syringyl and Guaiacyl lignin units in the cell wall. Higher S/G ratio is an indication of increase in saccharification efficiency [25] which is a desirable trait for bioethanol, bio-based and high value chemical production from lignocellulosic biomass. The differences in S/G ratio recorded in all the transgenic lines were significant when compared to control.

### Saccharification efficiency increased in transgenic poplar lines

**Fig. 5:**
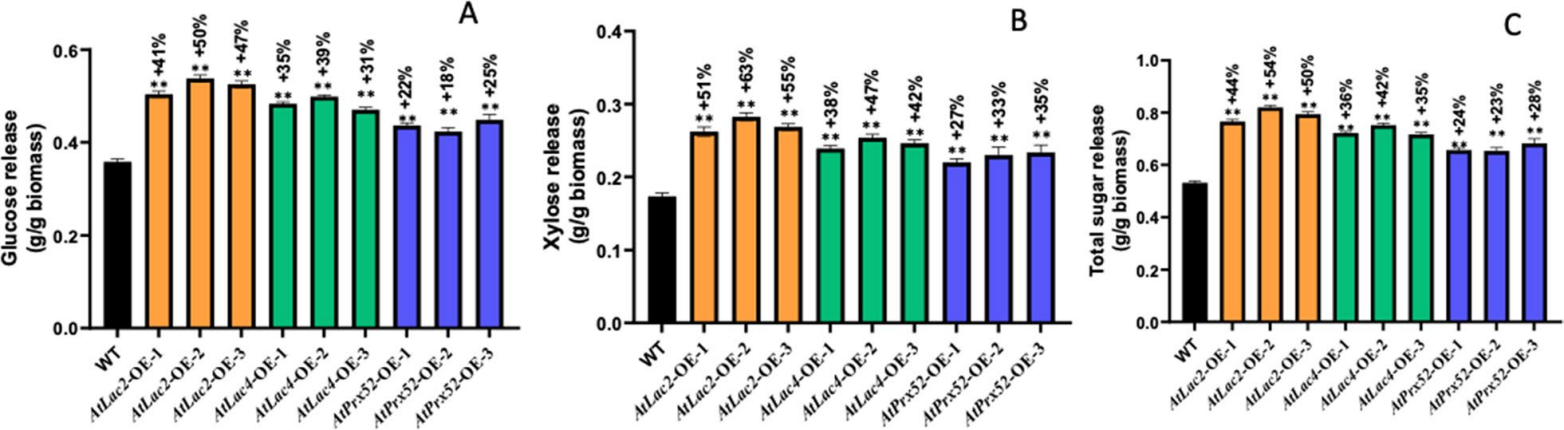
Saccharification efficiency measured for glucose release (**a**), xylose release (**b**), glucose release (**c**) total sugar release in each of the three transgenic lines for AtLac2-OE, AtLac4-OE, AtPrx-OE. Samples were measured in triplicate (*n* = 3). Significant differences were observed for the three transgenic lines. Significant as compared to wild type (*P* < 0.01)

Glucose release from transgenic poplar biomass expressing laccase and peroxidase was monitored by measuring absorbance at *620* nm. Hemicellulose and lignin were removed using acetonitrile treatment and crystalline cellulose was disordered upon treatment with sulfuric acid. Hence, monomeric glucose was available for colorimetric determination. Saccharification efficiency was significantly increased in all the three types of transgenic lines tested *AtLac2-OE, AtLac4-OE* and *AtPrx52-OE* for glucose and xylose release as well as total sugars for each of the candidate genes. In Fig. 5, transgenic lines showed 35–50% and 21–42% percent glucose release from lines *AtLac2-OE, and AtLac4-OE*, respectively. In case of *AtPrx52-OE*, increase in the percentage glucose release was 8–39% compared to non-transgenic controls. The increase in glucose release could be attributed to the change in lignin content and increase in S/G ratio which are likely responsible for introducing structural changes in the cell wall.

### Homology analysis of the Arabidopsis laccases genes

**Fig. 6:**
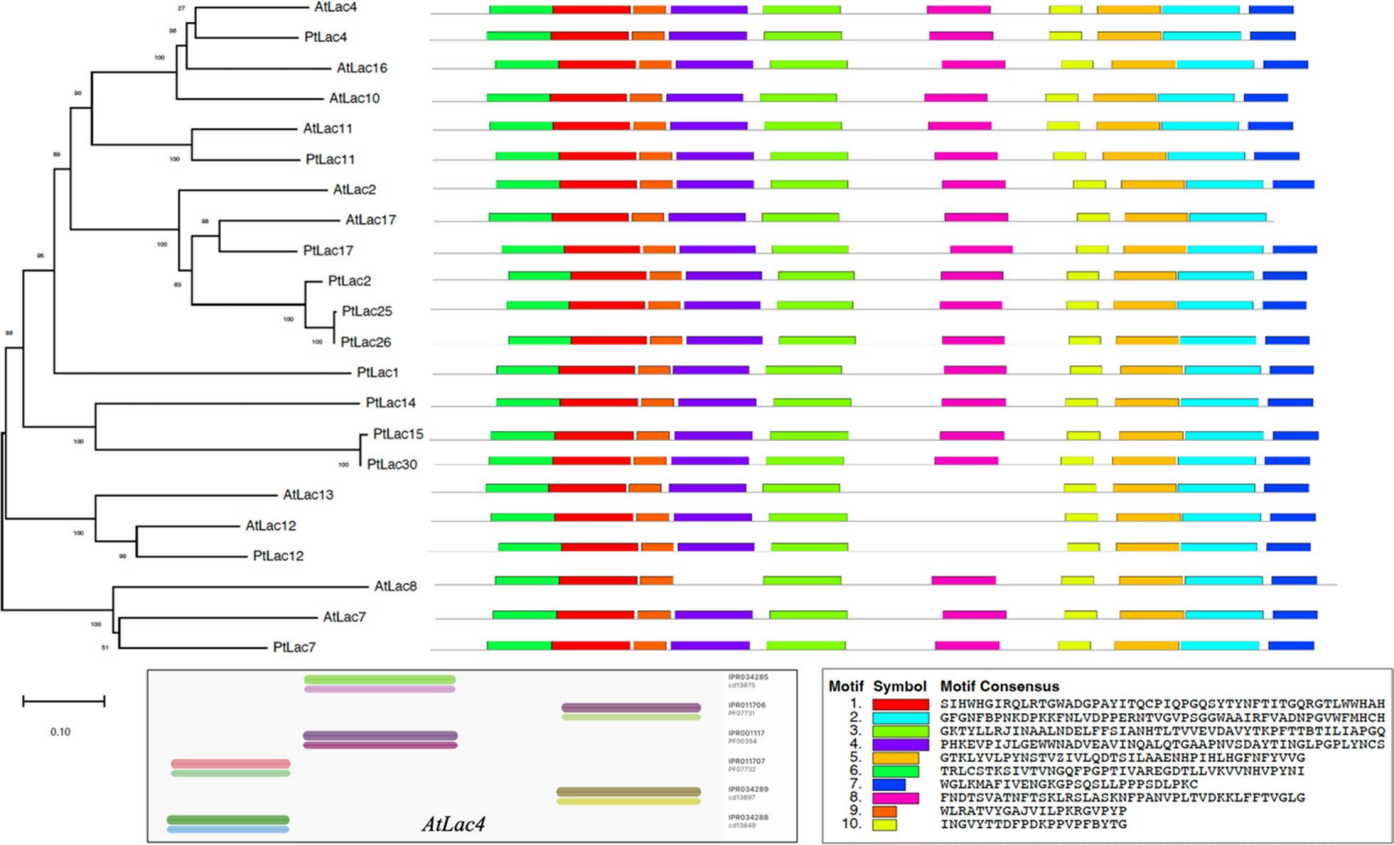
Phylogenetic relationship of laccases and their corresponding motif structures using full-length protein sequences obtained from BLASTP. The protein sequence of laccase in *A. thaliana* was used to analyze the domains using InterProScan

Homology analysis of Arabidopsis laccase genes involves the comparison of these genes to identify similarities and differences, which can provide insights into their evolutionary relationships and potential functional roles. Network analysis between AtLac2, AtLac4/ IRX12, and AtPrx52 will reveal the biological significance related to lignin biosynthesis. The BLAST and phylogenetics analysis is another bioinformatics analysis that should be described earlier as it shows the laccase homologs in poplar. It has the conserved domains between the two species assuming the species have similar functions. The laccase gene homologs in Arabidopsis showed more than 40% sequence identity with an e-value ranging from 2.79E-162 to 0.00 with the query sequence of AtLac4 against the Arabidopsis thaliana and Populus trichocarpa genomes (Additional file 2: Table S1). To elucidate the evolutionary relationship among the laccase genes in *Arabidopsis thaliana* and *Populus trichocarpa*, a phylogenetic tree was constructed for the selected laccases of both species using MEGA version 11.0 using the Neighbor-Joining approach with 1000 bootstrap iterations. Motif identification revealed seven conserved motifs (Motifs 1, 2, 3, 5, 6, 9, and 10) present in all laccases, highlighting the conservation of the motifs in these species (Fig. 8). Motif 3 and Motif 9 contain the delta-aminolevulinic acid dehydratase (PS00169) based on the PROSITE database annotation using TomTom. Detailed analysis of the AtLac4 protein sequence was also conducted using InterPro (https://www.ebi.ac.uk/interpro/) and found three domains: multicopper oxidase, C-terminal (IPR011706); multicopper oxidase, second cupredoxin domain (IPR001117), and multicopper oxidase, N-terminal (IPR011707) shown in Fig. 6.

### Homology analysis of the peroxidase genes

**Fig. 7:**
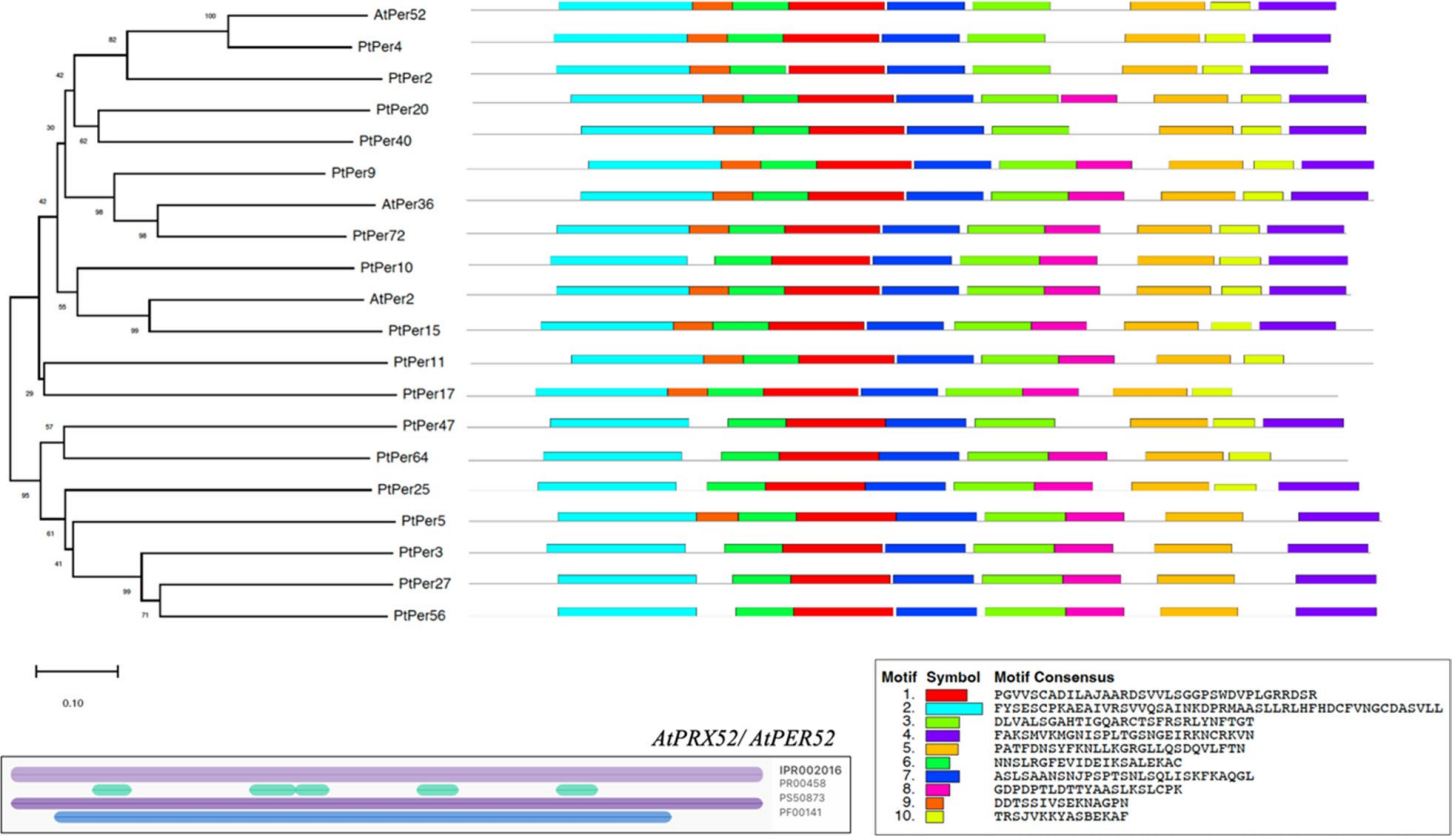
Phylogenetic relationship of peroxidases and their corresponding motif structures using full-length protein sequences obtained from BLASTP. The protein sequence of peroxidase (*AtPrx52*) in *A. thaliana* was used to analyze the domains using InterProScan

The peroxidase gene homologs in Arabidopsis showed more than 40% sequence identity with an e-value ranging from 6.41E-76 to 0.00 with the query sequence of *AtPrx52* against the *Arabidopsis thaliana* and *Populus trichocarpa* genomes (Additional file 2: Table S2). Next, a phylogenetic tree a phylogenetic tree was constructed for the selected peroxidases of *Arabidopsis thaliana* and *Populus trichocarpa* to elucidate the evolutionary relationship among the selected genes. Motif identification revealed six conserved motifs (Motifs 1, 2, 3, 5, 6, and 7) present in all peroxidases, highlighting the conservation of the motifs in these species (Fig. 9). Based on the motif distribution, the sequence of *AtPrx52* or *AtPer52* is more identical to the peroxidases from *Populus trichocarpa* in the similar clade (*PtPer4, PtPer2,* and *PtPer20*). Detailed analysis of the *AtPrx52* protein sequence was also conducted using InterPro (https://www.ebi.ac.uk/interpro/) and found domains and families related to peroxidases such as haem peroxidase (IPR002016) and secretory peroxidase (IPR033905) shown in Fig. 7.

### Gene network construction and GO enrichment analysis

**Fig. 8:**
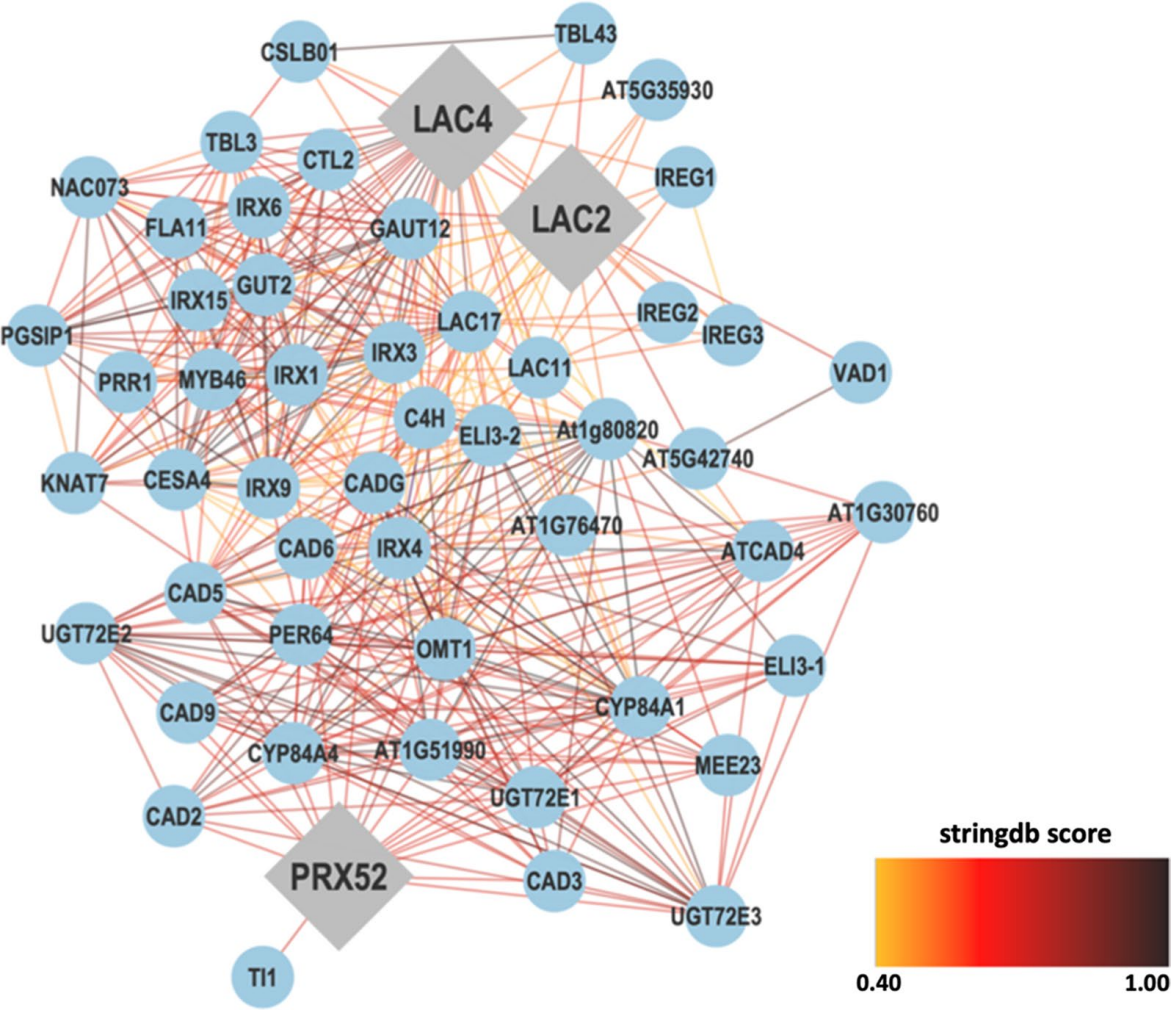
A gene network between *AtLac2*, *AtLac4,* and *AtPrx52* with 50 related genes and 441 interactions was obtained from the STRING database. The selected string score is 0.40 and above

**Fig. 9:**
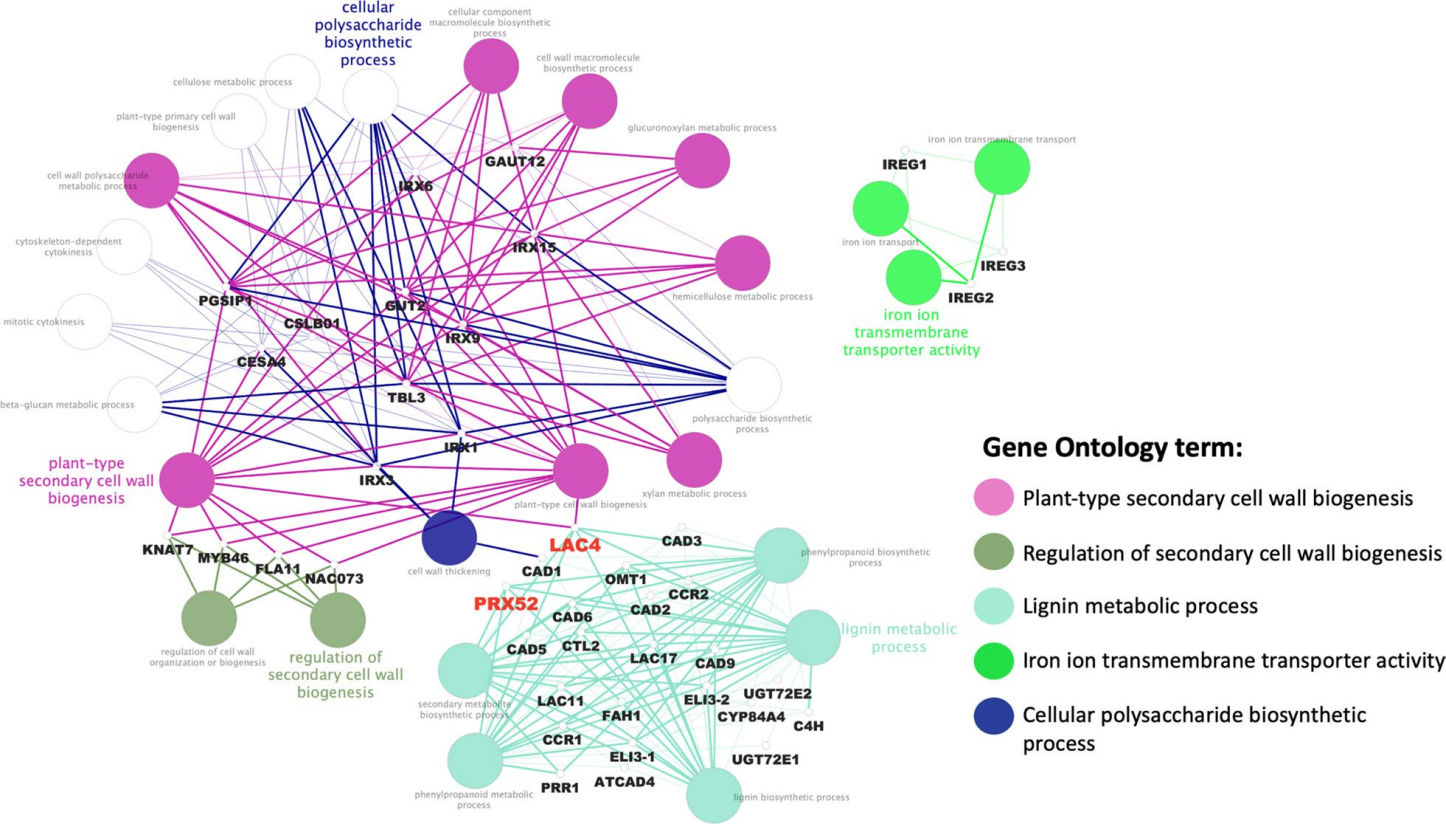
The GO enrichment using ClueGO/CluePedia apps from Cytoscape

*AtLac2*, *AtLac4*, and *AtPrx52* were used as bait genes to establish the interaction between genes using the STRING database. Figure 8 shows the interaction between the three query genes with 50 interacting partners, generating 441 interactions or edges. The gene network consisted of several functional categories related to the cellular polysaccharide biosynthetic process, regulation of cell wall biogenesis, phenylpropanoid pathway, and iron ion transmembrane transporter activity analyzed by ClueGO/ CluePedia (Fig. 9). Detailed information on the biological processes is listed in Additional file 2: Table S3.

### Statistical analysis

All experimental measurements and analysis were performed in triplicates. Three independent transgenic lines were considered for experimental study and data measurements. Figures for the data and statistical analysis were performed using Graphpad prism software (version 9.1). The significance was calculated using two-way ANOVA and significant differences observed were indicated by asterisks (*P < 0.05, **P < 0.01, ***P < 0.001).

## Discussion

Bioenergy production exploiting the biomass has emerged as a promising alternative solution to satiate the increasing global energy demands. However, economic production of bioethanol from lignocellulosic feedstocks depends on their use of saccharification, requiring chemical agents during pretreatments. Genetic modification of lignin synthesis has been shown to reduce feedstock recalcitrance for enzymatic saccharification [23]. The barrier to realising the potential of lignocellulosic bioethanol is understanding the recalcitrance of cellulosic biomass. Overcoming this biomass recalcitrance is the key challenge to large-scale production of lignocellulosic bioethanol [35]. Pretreatment is an important and critical step that enables enzyme hydrolysis of lignocellulose conversion to ethanol. Laccases and peroxidases play a role in the breakdown of lignin, which is a complex and rigid component of lignocellulosic biomass. Lignin acts as a barrier to the accessibility of cellulose and hemicellulose, the carbohydrate components that can be fermented to produce bioethanol [33]. Breaking down of phenolic polymer is essential to release these sugars and make them available for enzymes resulting into the fermentation of sugars. Laccases and peroxidases are involved in the oxidative degradation of lignin [31]. They can catalyze the breakdown of the complex lignin structure into smaller fragments. This breakdown of lignin facilitates the access of cellulolytic enzymes (e.g., cellulases) to cellulose and hemicellulose. Laccases and peroxidases can be used in combination with other enzymes to enhance the digestibility of enzymatic hydrolysis. The use of laccase and peroxidases in pretreatment processes can potentially reduce the number of cellulolytic enzymes required for efficient hydrolysis. This reduction in enzyme usage can contribute to cost-effectiveness in the bioethanol production process. Laccases and peroxidases are often used in combination with other pretreatment methods, such as steam explosion or acid pretreatment, to achieve a synergistic effect in lignin degradation and biomass conversion into biothanol. To enhance the saccharification in woody biomass species like poplars, we expressed AtLac2, AtLac4, AtPrx52 driven under developing xylem tissue-specific promoters in poplars. Primarily, peroxidases were considered as oxidative enzymes to be involved in lignin biosynthesis even before laccases were reported as key players in the polymerization of lignin monomer units [26]. However, later research on lignification also established connecting links between laccases and lignin biosynthesis [27]. Because of the multigene families encoding for laccases and peroxidases in plants, it is quite hard to ascertain the roles of each of these oxidative enzymes as they share a functional overlap among themselves. For example, in case of Prx2, 25 and 71 the oxidation sites for the substrates are Tyr74 and Tyr177 which is like CWPO-C, another cell wall peroxidase [28], therefore confirming their functions in lignification is yet not well understood. Role of laccases in lignification was confirmed in Arabidopsis stating that laccases are independent and functionally non-redundant in the plant vascular system [29].

It was reported that simultaneous disruption of lac11 with lac4, and lac17 genes in Arabidopsis completely blocked the plant growth, whereas casparian strips in endodermis were lignified in the triple mutant showing the activity of peroxidases in endodermis. Lignin content did not change in lac4 mutant but a significant decrease in lignin was observed in case of triple mutant lac4, lac11, lac17. This shows that both LAC and PRX are important for lignification in one or the other tissues and function independently. Functional redundancy was also observed in case of Populus where antisense suppression of individual Lac1, 3, 90, and 110 genes did not affect the lignin content [30]. Similarly, suppression of Lac2 in Populus was also reported to be playing a role in altering the cell wall chemistry without affecting the lignin content [31]. Our results indicated that expressing Arabidopsis LAC and PRX alters the cell wall chemistry by affecting lignin content and increasing S/G ratio. Lignin content was decreased in the transgenic lines and S/G ratio was significantly increased as compared to controls (Fig. 4). Without any chemical pretreatments, we observed a significant increase in the glucose release from the woody biomass obtained from these transgenic poplar lines.

**Fig. 10:**
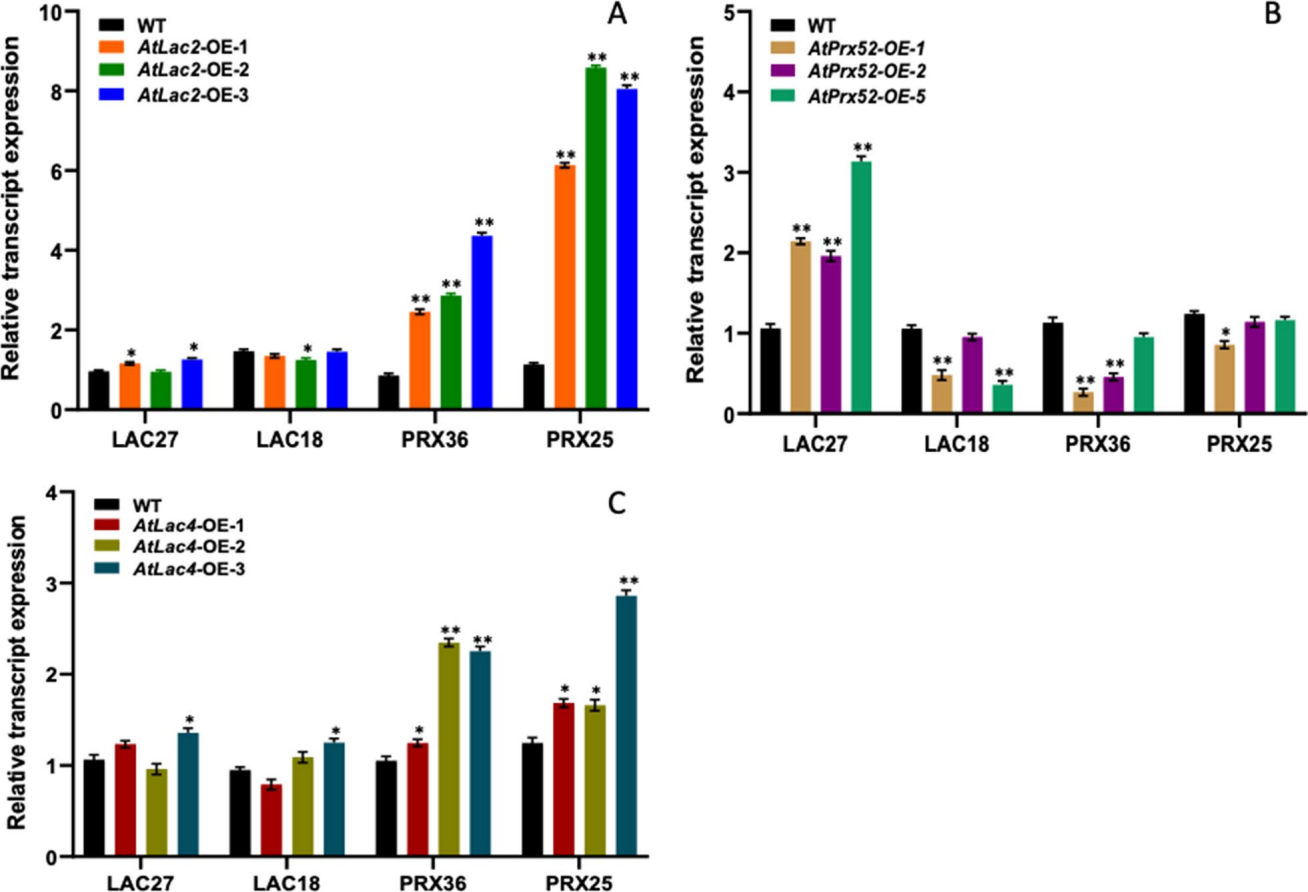
Relative transcript expression of selected genes like *Lac24, Lac18, Prx 36, Prx25* in the transgenic lines of AtLac2-OE (**A**), AtPrx-OE (**B**), AtLac4-OE (**C**). Samples were measured in triplicate (*n* = 3). Significant differences were observed for the three transgenic lines. Significant as compared to wild type (*P* < 0.01)

We investigated the transcript expression of some selected endogenous Lac and Prx genes in AtLac2-OE, AtLac4-OE and AtPrx52-OE lines. Endogenous candidate genes investigated in the poplar transgenic lines were Lac27, Lac18, Prx36 and Prx25 because these genes showed maximum expression in the stem differentiating xylem region, therefore we wanted to observe how expression of certain endogenous Lac and Prx genes was affected in transgenic poplars when we performed heterologous expression using AtLac2-OE, AtLac4-OE and AtPrx52-OE constructs in poplar. As shown in Fig. 6a–c, it was observed that in case of overexpression of AtLac2-OE and AtLac4-OE lines, endogenous poplar Prx36 and Prx25 were up-regulated and endogenous poplar Lac27 and Lac18 remained unchanged, whereas alteration in AtPrx52-OE resulted in up-regulation of endogenous Lac27 and native Prx36 and Prx25 remained unchanged (Fig. 10).

These findings supported previous observations that laccase and peroxidase functions are independent and non-redundant for maintaining the plant vasculature [29]. Sometimes function of LAC is compensated by PRX which was observed in case of triple mutant in Arabidopsis (lac4, 11, and 17) where plant growth was blocked by casparian strips showed lignin deposition through peroxidases. Exact functions of each member of these multigene families of LAC and PRX are still unclear. Thus, we carried out an extensive bioinformatics investigation to understand the roles of *AtLAC2*, *AtLAC4*, and *AtPRX52* through in silico analysis. The constructed gene network showed a relationship between *LAC4* and *PRX52* that are both involved in the lignin metabolic process (Fig. 9). *LAC4* is also involved in plant-type secondary cell wall biogenesis showing the capability of the enzyme in plant cell wall development and function. Co-expression analysis has been proven to be an effective and powerful means for the detection of functional gene modules underlying specific biological processes. We constructed the co-expression networks of genes and SCW biosynthetic-related genes that were involved in the regulation of SCW thickening. The results showed that Lac2, Lac4 and Prx52 were directly involved in the cellulose and hemicellulose biosynthesis genes and also involved in the direct control of phenylpropanoid pathway (Figs. 8 and 9). These results indicated that these Laccases and PRX are important regulators in the biosynthesis of specific SCW components. However, sometimes laccases and peroxidases show synergistic relationship in the biosynthesis of different cell wall components, indicating that these TFs and phenyl radicals might synergistically regulate the biosynthesis of different SCW components.

## Conclusions (future scope and limitations)

Most transgenic studies are conducted at either the in vitro level or green house level, however large-scale commercial production provides a true quantitative assessment for the study. Conventional breeding lags behind transgenic cultivars as transgenics provides higher yields and desired feedstock quality at a faster rate. Therefore, natural variations in the cultivars and selection of superior breeding cultivars in bioenergy crops based on transgenic trials seem to be viable options for building a better variety. There are number of ways to enhance the sugar by the classical breeding or transgenic development of biofuel crops in turn lowering the processing cost of the system. Conducted studies provide an additional possibility to modify a plant to obtain even higher saccharification. One of the major limitations is the cost associated with enzymatic and chemical pretreatments to scale up the industrial applications while maintaining cost-effectiveness strategy remains a challenge.

In context to Lac’s and Prx’s, we have reports stating how monolignols are synthesized through phenylpropanoid pathway, however, we do not have clear insights into polymerization of monolignols into lignin. LAC and PRX have been studied in a few plant systems but there are still many missing links as to which ones are more important in lignin polymerization. Currently, functions of a few LAC and PRX genes have been revealed in some plant species. Therefore, the presented study employed the overexpression of selected Lac’s and Prx enzymes in poplar to observe enhanced saccharification in the woody biomass. Expression profiling of LAC’s and PRX’s showing xylem tissue-specific expression could provide insights on their functions regarding lignification and saccharification. In future, formation of lignin structures in vivo needs to be studied to obtain some concrete ideas about its relation to other cell wall components so that deconstruction of cell wall will be easier. The conducted study demonstrated a potential approach that heterologous expression of some specific Arabidopsis LAC and PRX genes in poplars could be a good strategy for production of improved biomass with higher saccharification efficiency without impacting the plant growth and biomass.

## Materials and methods

### Vector construction, cloning and plant transformation

Two laccase genes from arabidopsis, namely, *Lac2* and *Lac4* were chosen as the candidate genes of interest out of 17 laccase genes in Arabidopsis based on their inflorescence-specific expression. *Prx52* from a total of 9 xylem-specific peroxidase genes was chosen as the other candidate gene. All the three candidate genes demonstrated significant transcript expression in the stem differentiating xylem based on the phylogenetic analysis and expression studies using Arabidopsis *Bar eFP* browser. The full-length cDNAs for *AtLac2, AtLac4* and *AtPrx52* were amplified using gene-specific primers) by thermocycler (Applied Bio-systems, USA). The PCR amplified products from the cDNAs of three candidate genes were eluted and subsequently digested with *XbaI* and *SacI* restriction enzymes. *DX15* promoter was cloned in the binary vector pBI101 using *HindIII* and *SalI* and GUS gene in *DX15:pBI101* binary vector was replaced with the cDNAs of interest.

### RNA extraction and gene expression studies through RT-PCR

Total RNA was isolated from stem differentiating xylem (SDX) taken from 20 to 50 internode from the base of plants using Trizol (Ambion, life Technologies) method. Extracted RNA was given DNase treatment (Turbo DNA free, ThermoFisher, USA). First strand cDNA synthesis was performed using 1 μg of total RNA using reverse transcription kit (Applied Bio-systems). Real time primers were designed for *AtLac2* (Gene ID-At2g29130), *AtLac4* (Gene ID-At2g38080) and *AtPrx52* (Gene ID-At5g05340) using Integrated DNA Technologies (IDT) software. Total reaction was 12 μl for each gene in triplicate with thermocycler conditions as: 95 °C for 10 min, 45 cycles for 95 °C for 30 s, 60 °C for 30 s. Relative gene expression was calculated by ΔΔCT method. *Actin7* was used as an internal control.

### Microscopic studies

For histology, sections were cut at 7th internode from the apex of poplar plants carrying around 50 internodes and preserved in ice-cold FAA (37% formaldehyde, acetic acid and 95% ethanol). The internodes were infiltrated through vacuum for about 10–15 min and kept overnight at 4 °C. Dehydration and embedding in wax was done for fixing the sections on slides. Dewaxing was done using two washes of xylene and decreasing concentrations of ethanol (95%, 80%, 75%, 60%) followed by washing in water solution. Autofluorescence was performed from dewaxed sections using fluorescent light microscope.

### Determination of laccase and peroxidase activity

Peroxidase activity was measured for SDX samples *AtPrx52-OE* transgenic lines and control plants using methods of [32] with certain modifications. One gram of SDX was homogenized in 5 ml of ice-cold extraction buffer (100 mM Tris–HCL, 100 mM NaHCO3, 10 mM MgCl2, 0.1 mM Na-EDTA, 10 mM 2-mercaptoethanol, 0.1% (*v*/*v*) Triton X-100) at 40 °C. After centrifugation at 25,000 g, supernatant was passed through sephadex G25 (Sigma-Aldrich) column for the protein purification. Eluate was taken in triplicate and used for the enzyme assay. Enzyme assay included potassium phosphate buffer pH 5.25, protein extract and hydrogen peroxide using guaiacol as substrate, measured at 436 nm for a time-period of 0–5 min. Laccase activity was performed for *AtLac2-OE* and AtLac4-OE transgenic plants following [33] and [34]. One gram of SDX was powdered in liquid nitrogen and homogenized in 10 ml of ice-cold extraction buffer (25 mM Tris pH 7, 200 mM CaCl2, 10% (*v*/*v*) glycerol, 4 µM sodium cacodylate, 1 mM PMSF, 1/200 (*v*/*v*) protease inhibitor cocktail (Sigma-Aldrich). After centrifugation, the supernatant was passed through column chromatography using concanavalin-A sepharose beads. Eluate was collected in triplicate and enzyme assay was performed at 420 nm using ABTS oxidation method.

### Lignin analysis using PyMBMS

After scrapping the developing xylem, the wood was dried at room temperature for two weeks and then milled to 20 mesh. One gram of wood powder was stored in falcon tubes and only 100 mg was used for lignin analysis. Three samples from each of the wood samples (belonging to independent transgenic lines) were sent to National Renewable Energy Laboratory (NREL) for analyses using Pyrolysis-Molecular Mass Beam Spectrometry (PyMBMS). Duplicate samples were used for each line to perform lignin analysis. Crissa Doeppke and Anne Ware from NREL, Colorado, performed the lignin analysis. Lignin analysis was done to calculate the lignin content (in percentage) and S/G ratio.

### Saccharification assay

Pretreatment and enzymatic hydrolysis of biomass was carried out as described 196 previously with some modifications [35]. Before the 197 analyses, biomass was treated with alpha-amylase (0.47 U per mg biomass, Sigma 198 Cat # A6255) in 100 mM ammonium formate (pH 5.0) buffer at 25 °C for 48 h to 199 remove starch, followed by three water and two acetone washes. Then biomass 200 samples were kept under the hood for 72 h for drying. This was followed by 201 ethanol Soxhlet extraction for an additional 24 h to remove extractives. After drying 202 overnight, pretreatment was carried out with 5 mg of dry biomass at 180 °C for 17.5 203 min. About 40 μl of buffer-enzyme stock, 8% CTec2 (Novozymes) in 1.0 M sodium 204 citrate buffer, was added to the pretreated biomass. The samples were incubated at 205 50 °C for 70 h. After 70 h of incubation, the hydrolysate was analyzed using 206 Megazyme’s GOPOD and XDH assays as described earlier.

### Homology analysis

The protein sequences of laccase and peroxidase genes in Arabidopsis were obtained from the UniProt database. Next, BLASTP was performed against the NCBI non-redundant protein sequences (nr), specifying only *Arabidopsis thaliana* (taxid:3702) and *Populus trichocarpa* (taxid:3694) to uncover the homologs between the two plant species. The selected homolog sequences from BLASTP hits were used to create a multiple sequence alignment using T-COFFEE (https://www.ebi.ac.uk/Tools/msa/tcoffee/) followed by phylogenetic tree construction in MEGA version 11.0 using the Neighbor-Joining technique with 1000 bootstrap iterations. Additionally, using Multiple Expectation Maximisation for Motif Elicitation (MEME) version 5.4 [36] the conserved motifs of the laccase and peroxidase genes from *A. thaliana* and *P. trichocarpa* were examined according to the following parameters: site distribution was set to zero or one occurrence (zoops), the maximum number of motifs for searching was set to 10, and the motif width was set between 6 and 50. Additionally, the PROSITE web server's ScanProsite interface was used to identify the conserved motifs for these genes [37].

### Gene network construction and GO enrichment analysis

In this study, a gene network was constructed using the Gene IDs of laccase and peroxidase genes in Arabidopsis; *AtLac2* (Gene ID-At2g29130), *AtLac4* (Gene ID-At2g38080) and *AtPrx52* (Gene ID-At5g05340). First, the Gene IDs were used as queries against the STRING database via Cytoscape and StringApp [38]. STRING is a biological network database that stores known and predicted interactions obtained from primary databases and computational approaches, respectively [39]. Next, we evaluate the biological role of the generated network using ClueGO/ CluePedia [40] apps in Cytoscape. Finally, a hypergeometric test with Bonferroni correction was used to compute the false discovery rate of each pathway to assess its significance.

### Supplementary Information

**Additional file 1.** BLASTP hits of AtLac2 against nr database specifying in *Arabidopsis thaliana* and *Populus trichocarpa*.

**Additional file 2: Table S1.** BLASTP hits of *AtLac4 *against nr database specifying in *Arabidopsis thaliana *and *Populus trichocarpa*. **Table S2.** BLASTP hits of *AtPrx52 *against nr database specifying in *Arabidopsis thaliana *and *Populus trichocarpa*. **Table S3.** Biological processes related to the gene network constructed by *AtLac2*, *AtLac4*, and *AtPrx52*.

## Data Availability

Wood material for the transgenic lines can be made available upon request.
