# Supplementary material for: Heterologous expression of Arabidopsis laccase2, laccase4 and peroxidase52 driven under developing xylem specific promoter DX15 improves saccharification in populus
Source: Biotechnol Biofuels Bioprod. 2024 Jan 13;17:5. doi: 10.1186/s13068-023-02452-7 (PMC10787383; doi:10.1186/s13068-023-02452-7)
Supplement: Supplementary file 1 — Additional file 1. BLASTP hits of AtLac2 against nr database specifying in Arabidopsis thaliana and Populus trichocarpa. [file 13068_2023_2452_MOESM1_ESM.pptx]

## Slide 1
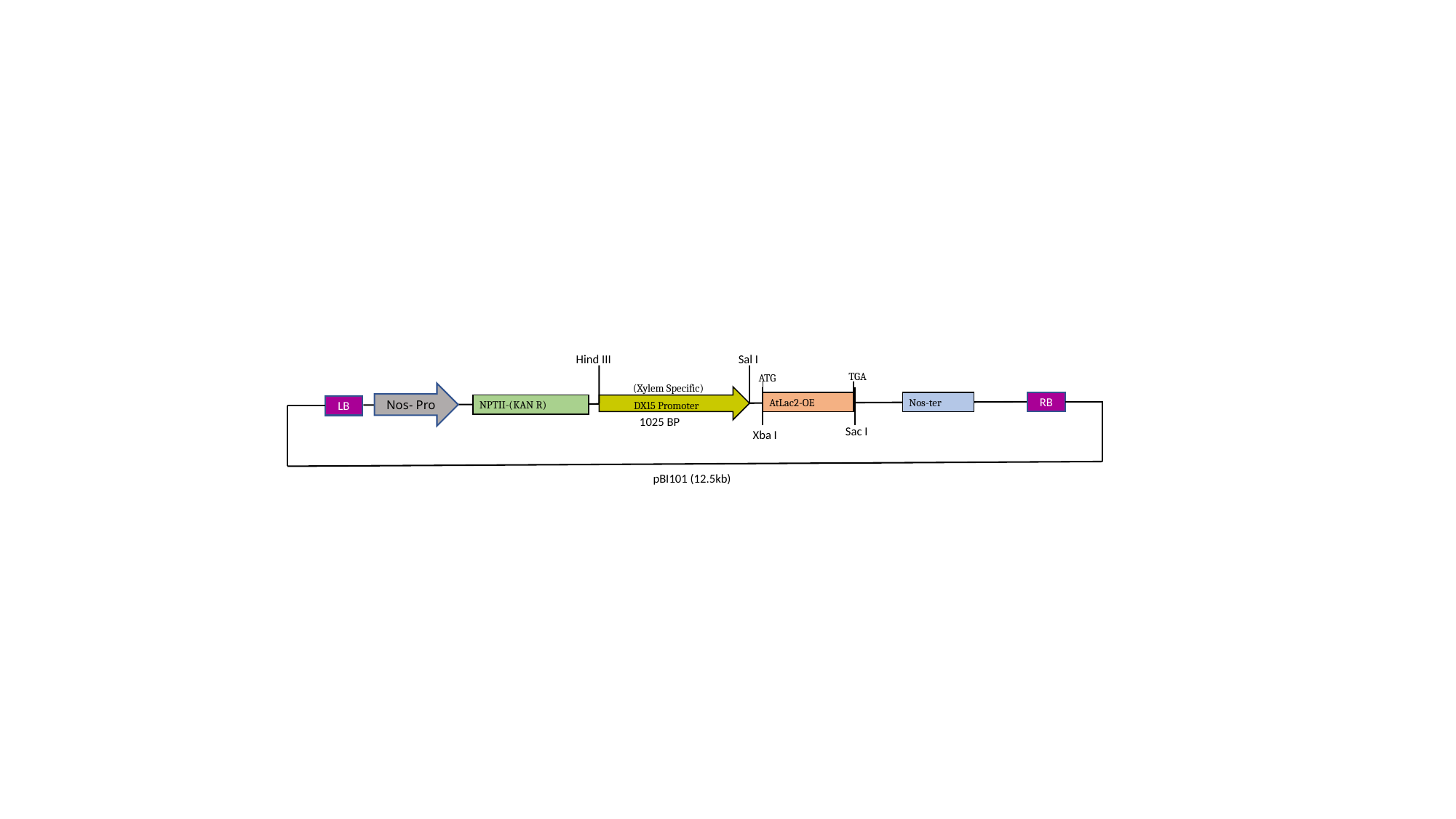

Hind III
Sal I
TGA
ATG
(Xylem Specific)
DX15 Promoter
AtLac2-OE
Nos-ter
NPTII-(KAN R)
Nos- Pro
RB
LB
1025 BP
Sac I
Xba I
pBI101 (12.5kb)

## Slide 2
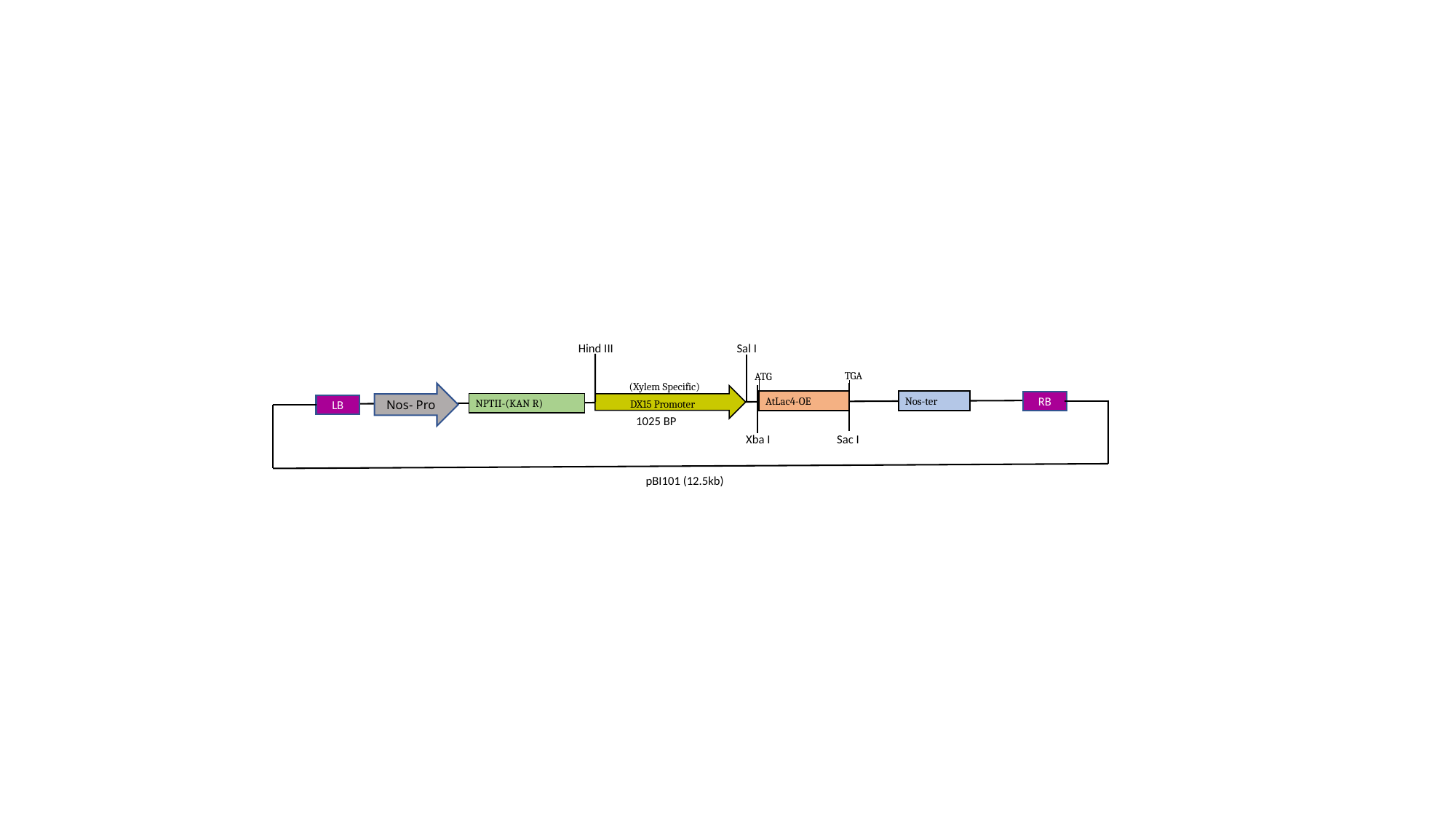

Hind III
Sal I
TGA
ATG
(Xylem Specific)
DX15 Promoter
AtLac4-OE
Nos-ter
NPTII-(KAN R)
Nos- Pro
RB
LB
1025 BP
Xba I
Sac I
pBI101 (12.5kb)

## Slide 3
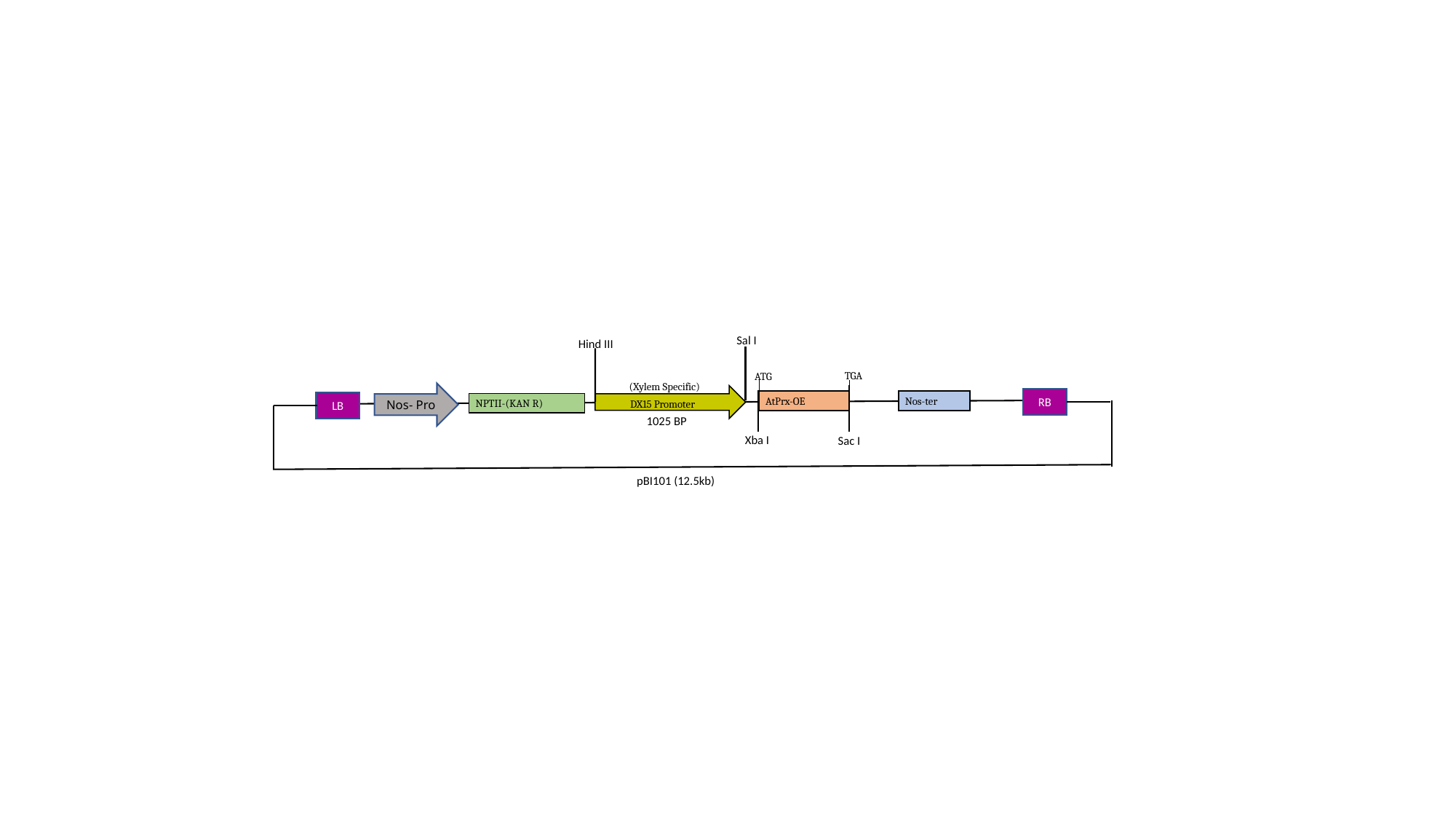

Sal I
Hind III
TGA
ATG
(Xylem Specific)
DX15 Promoter
AtPrx-OE
Nos-ter
NPTII-(KAN R)
Nos- Pro
RB
LB
1025 BP
Xba I
Sac I
pBI101 (12.5kb)

## Slide 4
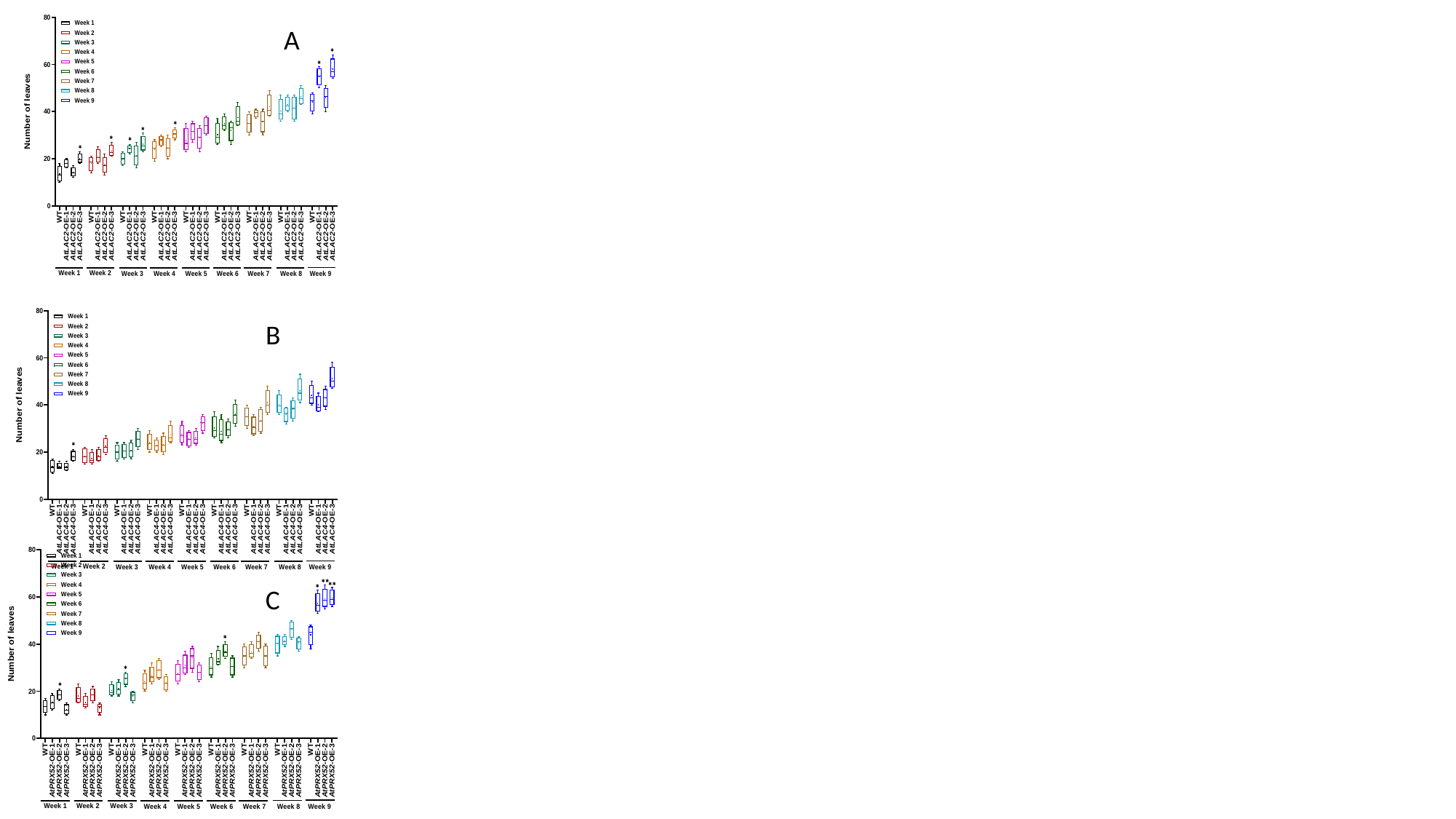

A
B
C

## Slide 5
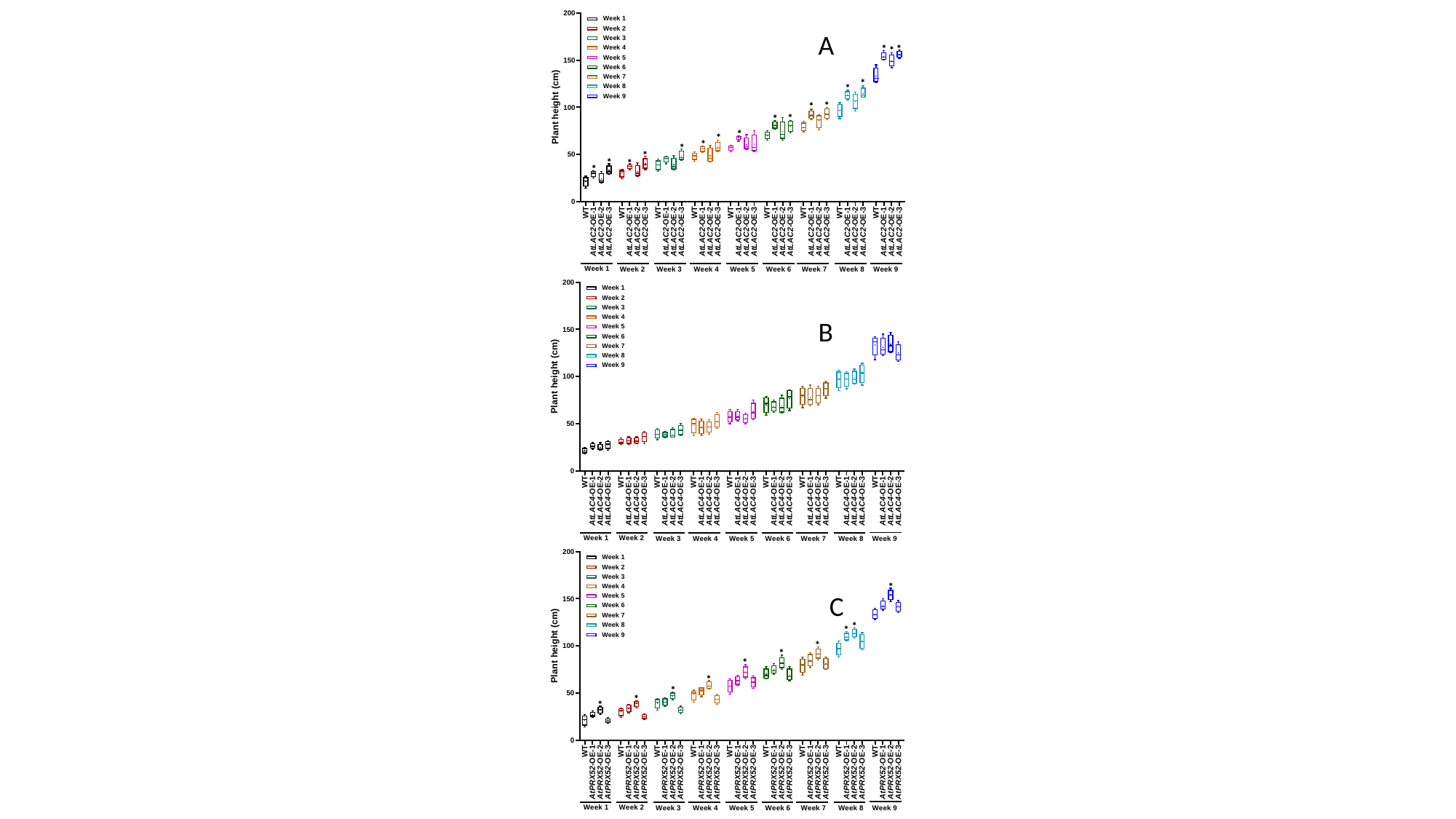

A
B
C

## Slide 6
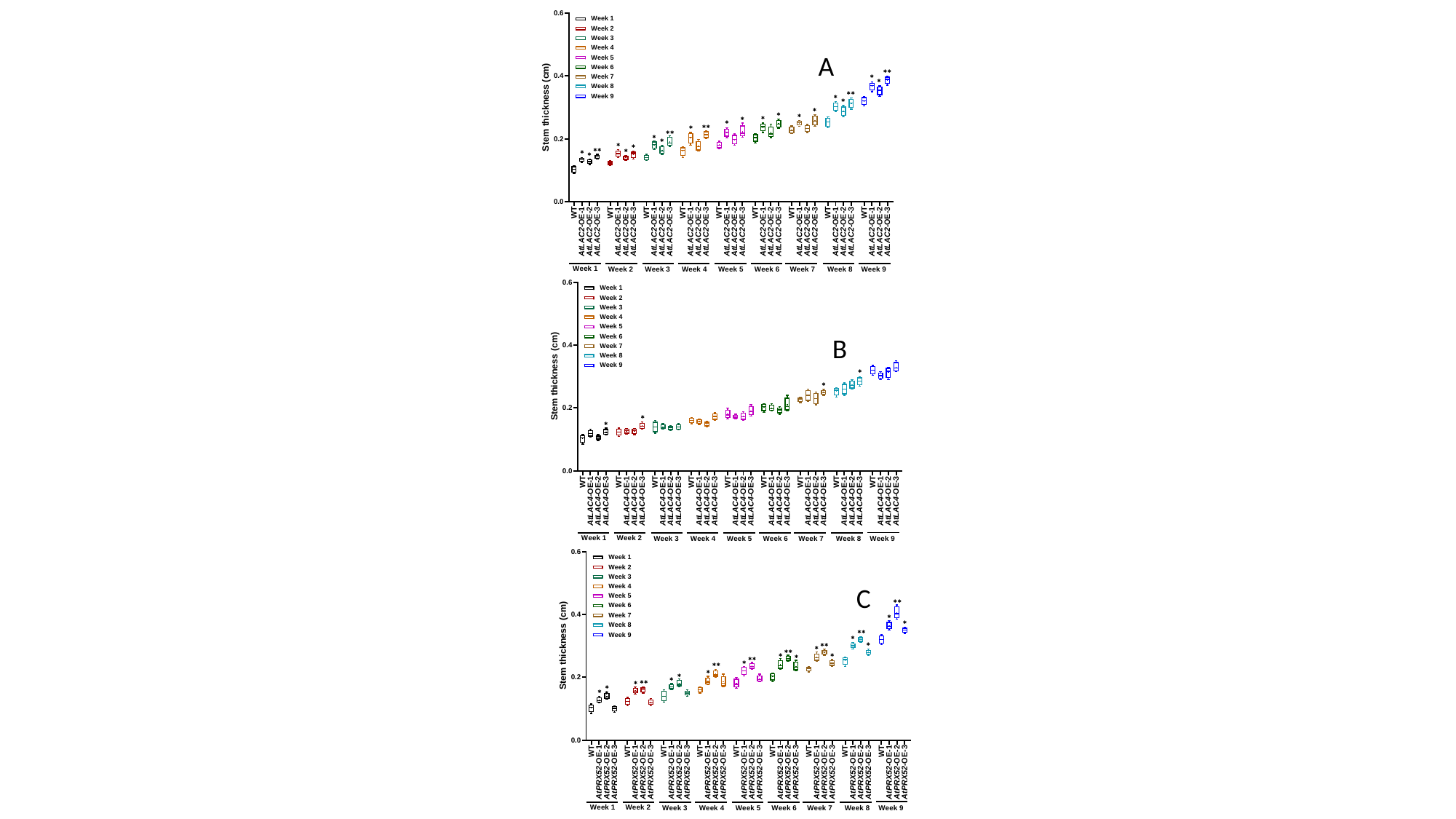

A
B
C

## Slide 7
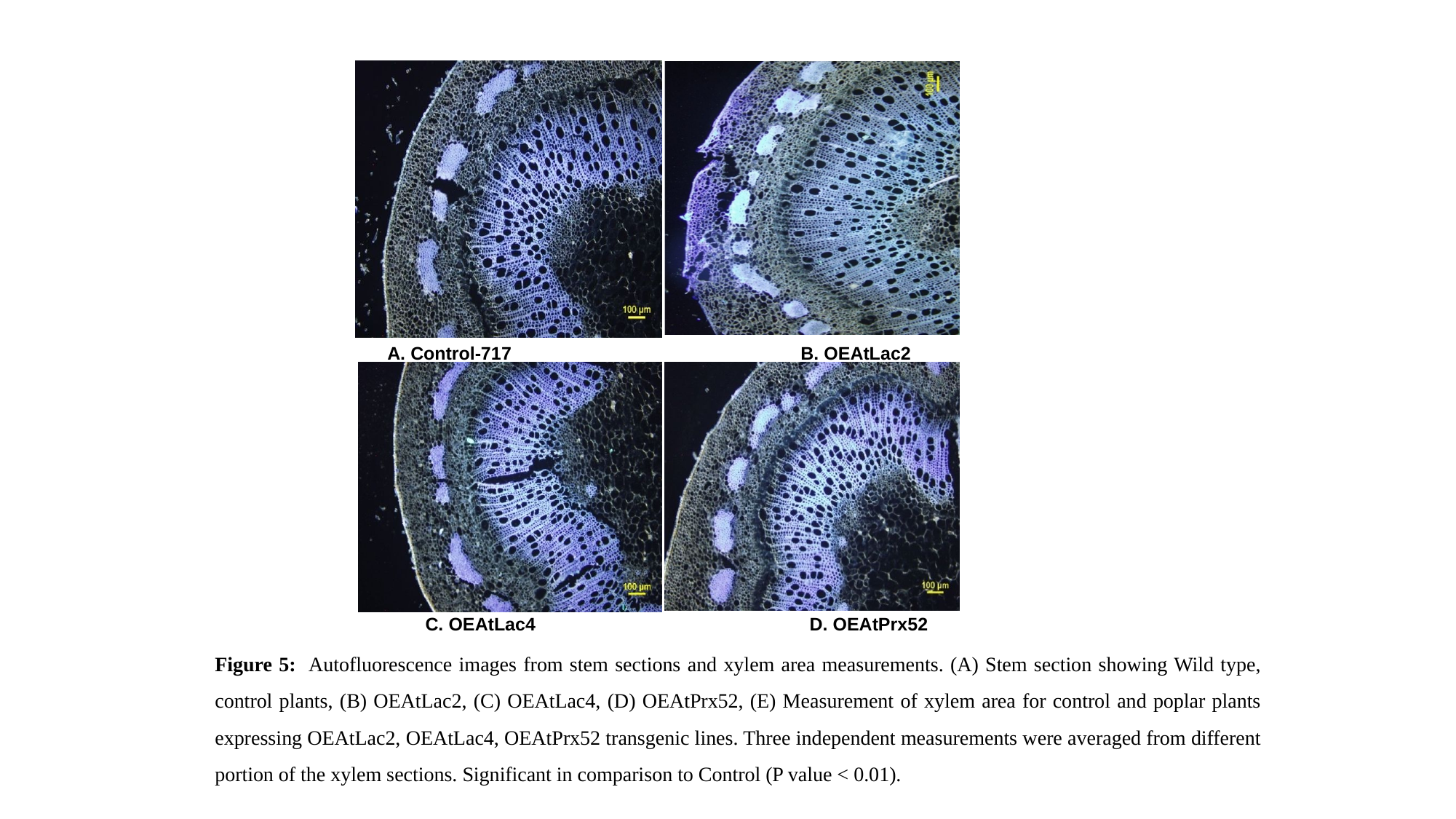

A. Control-717 B. OEAtLac2
C. OEAtLac4 D. OEAtPrx52
Figure 5: Autofluorescence images from stem sections and xylem area measurements. (A) Stem section showing Wild type, control plants, (B) OEAtLac2, (C) OEAtLac4, (D) OEAtPrx52, (E) Measurement of xylem area for control and poplar plants expressing OEAtLac2, OEAtLac4, OEAtPrx52 transgenic lines. Three independent measurements were averaged from different portion of the xylem sections. Significant in comparison to Control (P value < 0.01).
