# Supplementary material for: Heterologous expression of Arabidopsis laccase2, laccase4 and peroxidase52 driven under developing xylem specific promoter DX15 improves saccharification in populus
Source: Biotechnol Biofuels Bioprod. 2024 Jan 13;17:5. doi: 10.1186/s13068-023-02452-7 (PMC10787383; doi:10.1186/s13068-023-02452-7)
Supplement: Supplementary file 2 — Additional file 2: Table S1. BLASTP hits of AtLac4 against nr database specifying in Arabidopsis thaliana and Populus trichocarpa. Table S2. BLASTP hits of AtPrx52 against nr database specifying in Arabidopsis thaliana and Populus trichocarpa. Table S3. Biological processes related to the gene network constructed by AtLac2, AtLac4, and AtPrx52. [file 13068_2023_2452_MOESM2_ESM.docx]

**Additional file Tables**

## **Table S1** BLASTP hits of *AtLac4* against nr database specifying in *Arabidopsis thaliana* and *Populus trichocarpa.*

| **Non-redundant database** | **Gene Name/ID** | **Protein Name** | **Per. Ident (%)** | **Acc Length** | **E value** | **Species** |
| --- | --- | --- | --- | --- | --- | --- |
| NP_187533.1 | *LAC7* | laccase 7 | 46.355 | 550 | 2.79E-162 | *Arabidopsis thaliana* |
| NP_195739.2 | *LAC10* | laccase 10 | 74.014 | 558 | 0 | *Arabidopsis thaliana* |
| NP_001330402.1 | *LAC16* | laccase 16 | 70.87 | 567 | 0 | *Arabidopsis thaliana* |
| NP_195946.2 | *LAC11* | laccase 11 | 60.721 | 557 | 0 | *Arabidopsis thaliana* |
| NP_180477.1 | *LAC2* | laccase 2 | 54.594 | 573 | 0 | *Arabidopsis thaliana* |
| NP_196158.1 | *LAC12* | laccase 12 | 50.444 | 565 | 0 | *Arabidopsis thaliana* |
| NP_001330990.1 | *LAC17* | laccase 17 | 54.614 | 535 | 0 | *Arabidopsis thaliana* |
| NP_196330.3 | *LAC13* | laccase 13 | 49.209 | 569 | 0 | *Arabidopsis thaliana* |
| NP_195724.1 | *LAC8* | laccase 8 | 45.31 | 566 | 2.74E-164 | *Arabidopsis thaliana* |
| XP_002314124.1 | *LAC4* | laccase-4 | 76.854 | 556 | 0 | *Populus trichocarpa* |
| XP_002313847.4 | *LAC11* | laccase-11 | 59.075 | 561 | 0 | *Populus trichocarpa* |
| XP_002299296.1 | *LAC17* | laccase-17 | 59.683 | 581 | 0 | *Populus trichocarpa* |
| XP_002313424.1 | *LAC2* | laccase-2 | 55.342 | 576 | 0 | *Populus trichocarpa* |
| QZH55174.1 | *LAC26* | laccase 26 | 54.982 | 576 | 0 | *Populus trichocarpa* |
| QZH55173.1 | *LAC25* | laccase 25 | 54.982 | 576 | 0 | *Populus trichocarpa* |
| XP_002315131.2 | *LAC12* | laccase-12 | 50.823 | 576 | 0 | *Populus trichocarpa* |
| XP_002317883.1 | *LAC1* | laccase-1 | 48.448 | 579 | 0 | *Populus trichocarpa* |
| XP_002308196.1 | *LAC7* | laccase-7 | 49.468 | 562 | 0 | *Populus trichocarpa* |
| XP_024446768.2 | *LAC14* | laccase-14 | 47.689 | 566 | 0 | *Populus trichocarpa* |
| QZH55175.1 | *LAC30* | laccase 30 | 46.739 | 553 | 1.83E-166 | *Populus trichocarpa* |
| XP_006377535.2 | *LAC15* | laccase-15 | 46.558 | 557 | 7.87E-166 | *Populus trichocarpa* |

## **Table S2** BLASTP hits of *AtPrx52* against nr database specifying in *Arabidopsis thaliana* and *Populus trichocarpa.*

| **Non-redundant database** | **Gene Name/ID** | **Protein Name** | **Per. Ident (%)** | **Acc Length** | **E value** | **Species** |
| --- | --- | --- | --- | --- | --- | --- |
| XP_006376028.1 | *PER4* | peroxidase 4 | 73.75 | 322 | 1.76E-173 | *Populus trichocarpa* |
| XP_002323056.1 | *PER2* | peroxidase 2 | 53.918 | 321 | 2.88E-120 | *Populus trichocarpa* |
| NP_196290.1 | *PER2* | peroxidase 2 | 50.915 | 333 | 1.60E-108 | *Arabidopsis thaliana* |
| XP_006383152.1 | *PER72* | peroxidase 72 | 50.825 | 332 | 1.01E-106 | *Populus trichocarpa* |
| OAP06499.1 | *PER36* | peroxidase 36 | 51.148 | 341 | 5.72E-105 | *Arabidopsis thaliana* |
| XP_024461812.1 | *PER40* | peroxidase 40 | 51.827 | 337 | 1.66E-104 | *Populus trichocarpa* |
| XP_002306459.2 | *PER10* | peroxidase 10 | 48.366 | 322 | 5.84E-99 | *Populus trichocarpa* |
| XP_002300745.4 | *PER9* | peroxidase 9 | 48.355 | 345 | 8.24E-98 | *Populus trichocarpa* |
| XP_002304884.2 | *PER15* | peroxidase 15 | 46.429 | 329 | 1.38E-94 | *Populus trichocarpa* |
| XP_002315983.2 | *PER11* | peroxidase 11 | 43.235 | 340 | 1.90E-93 | *Populus trichocarpa* |
| XP_006384450.3 | *PER10* | peroxidase 10 | 47.119 | 339 | 2.64E-93 | *Populus trichocarpa* |
| XP_006368439.2 | *PER20* | peroxidase 20 | 46.278 | 337 | 1.18E-90 | *Populus trichocarpa* |
| XP_052303247.1 | *PER5* | peroxidase 5 | 42.727 | 324 | 2.82E-84 | *Populus trichocarpa* |
| XP_002308082.1 | *PER25* | peroxidase 25 | 42.136 | 321 | 1.74E-82 | *Populus trichocarpa* |
| XP_002310551.1 | *PER17* | peroxidase 17 | 43.377 | 320 | 8.71E-82 | *Populus trichocarpa* |
| XP_002304934.1 | *PER27* | peroxidase 27 | 41.317 | 327 | 2.32E-79 | *Populus trichocarpa* |
| XP_024448088.1 | *PER47* | peroxidase 47 | 42.761 | 316 | 3.71E-78 | *Populus trichocarpa* |
| XP_006372960.1 | *PER3* | peroxidase 3 | 41.83 | 324 | 3.47E-77 | *Populus trichocarpa* |
| XP_052310537.1 | *PER56* | peroxidase 56 | 40.789 | 327 | 2.85E-76 | *Populus trichocarpa* |
| XP_006383050.3 | *PER64* | peroxidase 64 | 41.391 | 317 | 6.41E-76 | *Populus trichocarpa* |

**Table S3** Biological processes related to the gene network constructed by *AtLac2*, *AtLac4*, and *AtPrx52*

| **ID** | **Term** | **Associated Genes Found** |
| --- | --- | --- |
| GO:1903338 | regulation of cell wall organization or biogenesis | [*FLA11, KNAT7, MYB46, NAC073*] |
| GO:2000652 | regulation of secondary cell wall biogenesis | [*FLA11, KNAT7, MYB46, NAC073*] |
| GO:0034755 | iron ion transmembrane transport | [*IREG1, IREG2, IREG3*] |
| GO:0006826 | iron ion transport | [*IREG1, IREG2, IREG3*] |
| GO:0005381 | iron ion transmembrane transporter activity | [*IREG1, IREG2, IREG3*] |
| GO:0044550 | secondary metabolite biosynthetic process | [*ATCAD4, C4H, CAD1, CAD2, CAD3, CAD5, CAD6, CAD9, CCR1, CCR2, CTL2, CYP84A4, ELI3-1, ELI3-2, FAH1, LAC4, LAC11, LAC17, OMT1, PRR1, PRX52*] |
| GO:0009698 | phenylpropanoid metabolic process | [*ATCAD4, C4H, CAD1, CAD2, CAD3, CAD5, CAD6, CAD9, CCR1, CCR2, CTL2, CYP84A4, ELI3-1, ELI3-2, FAH1, LAC4, LAC11, LAC17, OMT1, PRR1, PRX52, UGT72E1, UGT72E2*] |
| GO:0009699 | phenylpropanoid biosynthetic process | [*ATCAD4, C4H, CAD1, CAD2, CAD3, CAD5, CAD6, CAD9, CCR1, CCR2, CTL2, CYP84A4, ELI3-1, ELI3-2, FAH1, LAC4, LAC11, LAC17, OMT1, PRR1, PRX52*] |
| GO:0009808 | lignin metabolic process | [*ATCAD4, C4H, CAD1, CAD2, CAD3, CAD5, CAD6, CAD9, CCR1, CCR2, CTL2, CYP84A4, ELI3-1, ELI3-2, FAH1, LAC4, LAC11, LAC17, OMT1, PRX52, UGT72E1, UGT72E2*] |
| GO:0009809 | lignin biosynthetic process | [*ATCAD4, CAD1, CAD2, CAD3, CAD5, CAD6, CAD9, CCR1, CCR2, CTL2, CYP84A4, ELI3-1, ELI3-2, FAH1, LAC4, LAC11, LAC17, OMT1, PRX52*] |
| GO:0061640 | cytoskeleton-dependent cytokinesis | [*CESA4, CSLB01, IRX1, IRX3*] |
| GO:0000271 | polysaccharide biosynthetic process | [*CESA4, CSLB01, GAUT12, GUT2, IRX1, IRX15, IRX3, IRX6, IRX9, PGSIP1, TBL3*] |
| GO:0000281 | mitotic cytokinesis | [*CESA4, CSLB01, IRX1, IRX3*] |
| GO:0009833 | plant-type primary cell wall biogenesis | [*CESA4, CSLB01, IRX1, IRX3*] |
| GO:0033692 | cellular polysaccharide biosynthetic process | [*CESA4, CSLB01, GAUT12, GUT2, IRX1, IRX15, IRX3, IRX6, IRX9, PGSIP1, TBL3*] |
| GO:0052386 | cell wall thickening | [*CAD1, CESA4, IRX1, IRX3*] |
| GO:0051273 | beta-glucan metabolic process | [*CESA4, CSLB01, IRX1, IRX3, IRX6, TBL3*] |
| GO:0030243 | cellulose metabolic process | [*CESA4, CSLB01, IRX1, IRX3, IRX6, TBL3*] |
| GO:0051274 | beta-glucan biosynthetic process | [*CESA4, CSLB01, IRX1, IRX3, IRX6, TBL3*] |
| GO:0030244 | cellulose biosynthetic process | [*CESA4, CSLB01, IRX1, IRX3, IRX6, TBL3*] |
| GO:0009832 | plant-type cell wall biogenesis | [*CESA4, CSLB01, FLA11, GUT2, IRX1, LAC4, IRX15, IRX3, IRX6, IRX9, KNAT7, MYB46, NAC073, PGSIP1*] |
| GO:0061640 | cytoskeleton-dependent cytokinesis | [*CESA4, CSLB01, IRX1, IRX3*] |
| GO:0000271 | polysaccharide biosynthetic process | [*CESA4, CSLB01, GAUT12, GUT2, IRX1, IRX15, IRX3, IRX6, IRX9, PGSIP1, TBL3*] |
| GO:0000281 | mitotic cytokinesis | [*CESA4, CSLB01, IRX1, IRX3*] |
| GO:0010383 | cell wall polysaccharide metabolic process | [*GAUT12, GUT2, IRX15, IRX3, IRX6, IRX9, PGSIP1, TBL3*] |
| GO:0070589 | cellular component macromolecule biosynthetic process | [*GAUT12, GUT2, IRX15, IRX6, IRX9, PGSIP1, TBL3*] |
| GO:0009833 | plant-type primary cell wall biogenesis | [*CESA4, CSLB01, IRX1, IRX3*] |
| GO:0009834 | plant-type secondary cell wall biogenesis | [*CESA4, FLA11, GUT2, IRX1, LAC4, IRX15, IRX3, IRX6, IRX9, KNAT7, MYB46, NAC073, PGSIP1*] |
| GO:0033692 | cellular polysaccharide biosynthetic process | [*CESA4, CSLB01, GAUT12, GUT2, IRX1, IRX15, IRX3, IRX6, IRX9, PGSIP1, TBL3*] |
| GO:0044038 | cell wall macromolecule biosynthetic process | [*GAUT12, GUT2, IRX15, IRX6, IRX9, PGSIP1, TBL3*] |
| GO:0010410 | hemicellulose metabolic process | [*GAUT12, GUT2, IRX15, IRX9, PGSIP1, TBL3*] |
| GO:0051273 | beta-glucan metabolic process | [*CESA4, CSLB01, IRX1, IRX3, IRX6, TBL3*] |
| GO:0070592 | cell wall polysaccharide biosynthetic process | [*GAUT12, GUT2, IRX15, IRX6, IRX9, PGSIP1, TBL3*] |
| GO:0045491 | xylan metabolic process | [*GAUT12, GUT2, IRX15, IRX9, PGSIP1, TBL3*] |
| GO:0030243 | cellulose metabolic process | [*CESA4, CSLB01, IRX1, IRX3, IRX6, TBL3*] |
| GO:0045492 | xylan biosynthetic process | [*GAUT12, GUT2, IRX15, IRX9, PGSIP1, TBL3*] |
| GO:0051274 | beta-glucan biosynthetic process | [*CESA4, CSLB01, IRX1, IRX3, IRX6, TBL3*] |
| GO:0010413 | glucuronoxylan metabolic process | [*GAUT12, GUT2, IRX9, PGSIP1*] |
| GO:0010417 | glucuronoxylan biosynthetic process | [*GAUT12, GUT2, IRX9, PGSIP1*] |
| GO:0030244 | cellulose biosynthetic process | [*CESA4, CSLB01, IRX1, IRX3, IRX6, TBL3*] |
